# Japanese Encephalitis Virus Nonstructural Protein NS5 Interacts with Mitochondrial Trifunctional Protein and Impairs Fatty Acid β-Oxidation

**DOI:** 10.1371/journal.ppat.1004750

**Published:** 2015-03-27

**Authors:** Yu-Ting Kao, Bi-Lan Chang, Jian-Jong Liang, Hang-Jen Tsai, Yi-Ling Lee, Ren-Jye Lin, Yi-Ling Lin

**Affiliations:** 1 Graduate Institute of Life Sciences, National Defense Medical Center, Taipei, Taiwan; 2 Institute of Biomedical Sciences, Academia Sinica, Taipei, Taiwan; 3 Department of General Medicine, School of Medicine, College of Medicine, Taipei Medical University, Taipei, Taiwan; 4 Department of Primary Care Medicine, Taipei Medical University Hospital, Taipei, Taiwan; 5 Genomics Research Center, Academia Sinica, Taipei, Taiwan; University of Queensland, AUSTRALIA

## Abstract

Infection with Japanese encephalitis virus (JEV) can induce the expression of pro-inflammatory cytokines and cause acute encephalitis in humans. β-oxidation breaks down fatty acids for ATP production in mitochondria, and impaired β-oxidation can induce pro-inflammatory cytokine expression. To address the role of fatty-acid β-oxidation in JEV infection, we measured the oxygen consumption rate of mock- and JEV-infected cells cultured with or without long chain fatty acid (LCFA) palmitate. Cells with JEV infection showed impaired LCFA β-oxidation and increased interleukin 6 (IL-6) and tumor necrosis factor α (TNF-α) expression. JEV nonstructural protein 5 (NS5) interacted with hydroxyacyl-CoA dehydrogenase α and β subunits, two components of the mitochondrial trifunctional protein (MTP) involved in LCFA β-oxidation, and NS5 proteins were detected in mitochondria and co-localized with MTP. LCFA β-oxidation was impaired and higher cytokines were induced in cells overexpressing NS5 protein as compared with control cells. Deletion and mutation studies showed that the N-terminus of NS5 was involved in the MTP association, and a single point mutation of NS5 residue 19 from methionine to alanine (NS5-M19A) reduced its binding ability with MTP. The recombinant JEV with NS5-M19A mutation (JEV-NS5-M19A) was less able to block LCFA β-oxidation and induced lower levels of IL-6 and TNF-α than wild-type JEV. Moreover, mice challenged with JEV-NS5-M19A showed less neurovirulence and neuroinvasiveness. We identified a novel function of JEV NS5 in viral pathogenesis by impairing LCFA β-oxidation and inducing cytokine expression by association with MTP.

## Author Summary

Lipids are involved in various steps of viral infection, and viruses may alter lipid metabolism to facilitate efficient viral replication. To address whether long-chain fatty acid (LCFA) metabolism is affected by Japanese encephalitis virus (JEV) infection, the leading cause of viral encephalitis in Asia, we compared the oxygen consumption rate of mock- and JEV-infected cells cultured with or without LCFA. LCFA utilization was impaired in JEV-infected cells, and higher pro-inflammatory cytokine expression was induced when LCFA was the major energy source. JEV nonstructural protein 5 (NS5) interacted with mitochondrial trifunctional protein, an enzyme complex involved in LCFA β-oxidation, and the interaction impaired LCFA β-oxidation, enhanced cytokine production, and contributed to JEV pathogenesis. The M19 residue of NS5 is involved in its interaction with MTP and the recombinant JEV with NS5-M19A mutation was less able to block LCFA β-oxidation, induced lower levels of cytokine production and showed less neurovirulence and neuroinvasiveness than wild-type JEV. Thus, impaired LCFA β-oxidation and enhanced cytokine production induced by JEV NS5 may provide new insight into JEV virulence.

## Introduction

Japanese encephalitis virus (JEV) is a member of the *Flaviviridae* family of many important human pathogens such as yellow fever virus, dengue virus (DENV), West Nile virus (WNV), tick-borne encephalitis virus (TBEV) and hepatitis C virus (HCV) [1]. JEV is the leading cause of viral encephalitis in Asia, with more than 50,000 cases and 10,000 deaths annually [2,3]. The genome of JEV is a positive-sense RNA encoding a polyprotein that is proteolytically processed into three structural proteins (core [C], precursor of membrane [prM], and envelope protein [E]) and seven nonstructural proteins (NS1, NS2A, NS2B, NS3, NS4A, NS4B, and NS5). NS5 is the largest flaviviral protein, with enzymatic activities of methyltransferase (MTase) and RNA-dependent RNA polymerase (RdRP) required for viral replication [4–6]. Interferon (IFN) antagonistic roles have also been demonstrated for flaviviral NS5; for example, the NS5 proteins of JEV, WNV and TBEV can block IFN-triggered JAK-STAT signaling [7–9] and DENV NS5 can cause degradation of STAT2 protein [10].

Lipids are involved in various steps of viral infection, such as viral entry, RNA replication, virion assembly and energy supply, and viruses are known to modulate cellular lipid metabolism [11,12]. Fatty acids synthesized from acetyl-CoA by lipogenesis may serve as precursors to produce lipid components or be broken down for ATP production via β-oxidation [13]. Long-chain fatty acids (LCFAs) are transported into mitochondria with the help of carnitine, then β-oxidation splits LCFA into acetyl-CoA via a four-step reaction [14]. Three of the 4 enzymatic activities of β-oxidation are catalyzed by a protein complex called mitochondrial trifunctional protein (MTP), consisting of hydroxyacyl-CoA dehydrogenase subunit A and subunit B (HADHα and HADHβ) [14,15]. The hallmark of MTP deficiency is accumulation of long-chain 3-hydroxy fatty acids [16–18], which, trapped inside the mitochondrial matrix, induce reactive oxygen species (ROS) production and pro-inflammatory cytokine expression [19,20]. Blockage of LCFA β-oxidation may also increase glucose consumption and result in hypoglycemia [21,22], which is deleterious to the central nervous system (CNS) [23]. Thus, patients with MTP deficiency might have serious complications, with damaged organs and long-term irreversible neuropathic complications with progressive encephalopathy.

Positive-sense RNA viruses induce intracellular membrane rearrangements to create favorable sites for viral replication. To synthesize and reorganize the intracellular membranes, HCV increases *de novo* synthesis and uptake of fatty acids, and also inhibits β-oxidation [24,25]. These lipid modulations may lead to abnormal accumulation of fat deposits in the liver (steatosis) [26,27] and are associated with chronic inflammatory response, features commonly seen in HCV patients [28]. Impaired LCFA β-oxidation has been implicated in influenza-associated neuronal disease, because patients with fatal and handicapped influenza-associated encephalopathy showed increased serum acylcarnitine ratio of C_16:0_+C_18:0_ to C_2_ [29]. Acute Japanese encephalitis (JE), characterized by inflammatory mediators in the brain, can develop in humans stung by a JEV-infected mosquito. The neurological dysfunction caused by activated immune cells is via induction of pro-inflammatory cytokines and ROS production, which leads to increased permeability of the blood brain barrier [30–32]. The levels of tumor necrosis factor α (TNF-α) and interleukin 6 (IL-6) were elevated in serum and cerebrospinal fluid of JE patients, with their induction associated with fatal outcome of JE [33,34].

Despite the link between lipid metabolism and inflammation in viral diseases, the role of fatty acid metabolism in JEV infection is largely unknown. In this study, we addressed whether LCFA β-oxidation is modulated by JEV infection and its potential involvement in JEV pathogenesis. We further studied the molecular mechanism of how JEV hinders cellular β-oxidation and found that JEV NS5 interacts with HADHα and HADHβ, two subunits of the enzyme complex MTP involved in LCFA β-oxidation. Furthermore, the recombinant JEV carrying a mutated NS5, with less binding ability with MTP, was less able to block LCFA β-oxidation, triggered reduced cytokine production, and featured less virulence. We discuss the novel function of JEV NS5 in modulating LCFA β-oxidation and cytokine induction.

## Results

### Impaired LCFA β-oxidation induces cytokine production in JEV-infected cells

**Fig 1:**
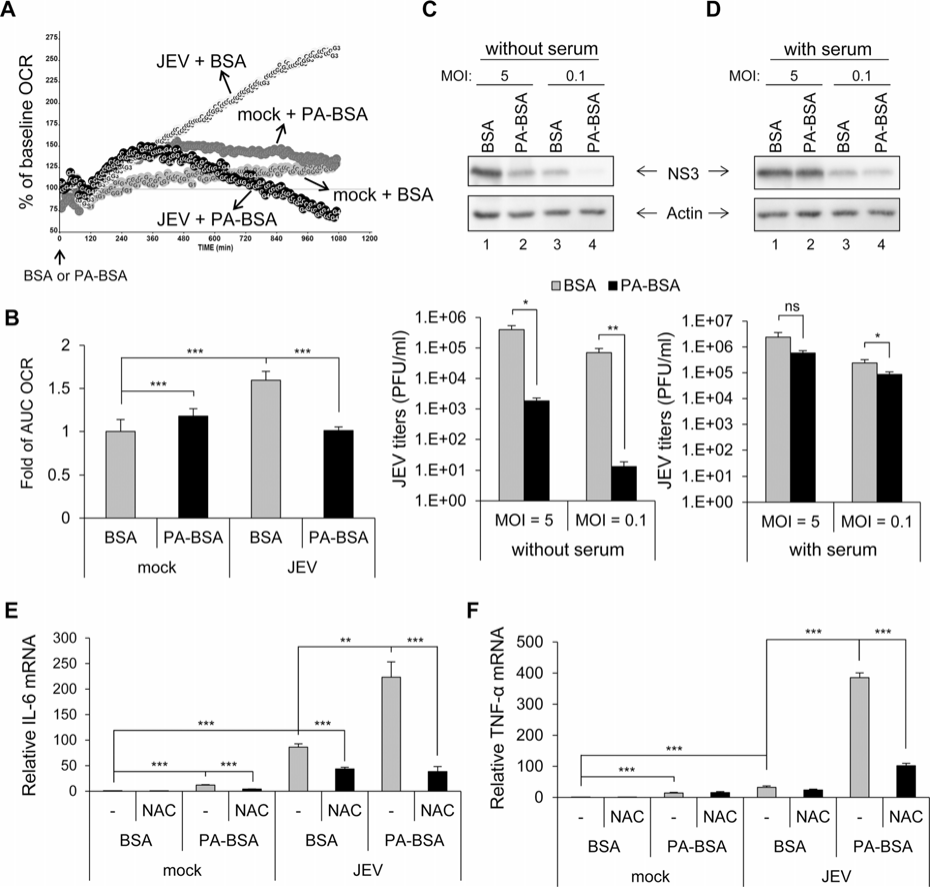
Impaired long-chain fatty acid (LCFA) β-oxidation and induction of reactive oxygen species (ROS)-dependent pro-inflammatory cytokines in cells infected with Japanese encephalitis virus (JEV). (A and B) A549 cells infected with JEV (multiplicity of infection [MOI] = 10) for 5 h were replenished with serum-free medium for 1 h, then treated with 200 μM palmitate conjugated to bovine serum albumin (BSA) (PA-BSA) or BSA control. (A) Real-time oxygen consumption rate (OCR) measured from 6 to 24 h post-infection (hpi). The OCR before PA-BSA or BSA treatment was set to 100%. (B) The area under the curve (AUC) OCR compared to that for mock cells treated with BSA (n = 3 per group). (C and D) A549 cells infected with JEV (MOI = 5 and 0.1) for 5 h were changed to medium without serum (C) or with serum (10% FBS) (D) for 1 h. Cells were then treated with PA-BSA or BSA for 18 h before Western blot analysis of protein levels of JEV NS3 and actin in cell lysates and virus titration in culture supernatants by plaque-forming assay (n = 3). (E and F) A549 cells were treated with N-acetylcysteine (NAC) 1 h before JEV (MOI = 10) infection and after virus adsorption. At 5 hpi, cells were incubated with serum-free medium for 1 h before treatment with PA-BSA or BSA for 18 h. RT-qPCR analysis of the relative mRNA levels of interleukin 6 (IL-6) (E) and tumor necrosis factor α (TNF-α) (F) (n = 3). Data are mean±SD. *P < 0.05, **P < 0.01, ***P < 0.001 and ns, not significant.

Palmitic acid is the most common fatty acid in animals, so we and others have been using sodium palmitate conjugated bovine serum albumin (PA-BSA) to study fatty acid β-oxidation [35]. During fasting, fatty acid oxidation becomes the major energy source [36,37] and oxygen consumption will be mainly resulted from β-oxidation in cells cultured with PA-BSA under starvation condition (without serum). To address whether JEV infection modulates cellular LCFA β-oxidation, we measured the oxygen consumption rate (OCR) in cells cultured with a noncytotoxic dose of PA-BSA (S1 Fig) or BSA control by using a metabolic XF24 analyzer [35]. In JEV-infected human A549 cells cultured with BSA, OCR values continued to increase from 6 to 24 h post-infection (hpi; Fig. 1A, JEV + BSA). However, in JEV-infected cells cultured with PA-BSA, the OCR values increased in the beginning, then decreased from about 11 hpi until the end of the recording (Fig. 1A, JEV + PA-BSA). Mock-infected cells did not show the distinct OCR patterns with BSA and PA-BSA treatments (Fig. 1A). Changes in OCR values represented by area under the curve (AUC) similarly showed that the AUC OCR was lower in JEV-infected A549 cells cultured with PA-BSA than BSA (Fig. 1B). This phenomenon was not limited to a single cell type and also occurred in JEV-infected human neuroblastoma HTB-11 cells cultured with PA-BSA or BSA (S2A Fig). Thus, the reduced OCR in JEV-infected cells cultured with palmitate indicates that JEV cannot utilize LCFA efficiently, probably because of blocked β-oxidation.

We then assessed the effect of impaired β-oxidation on JEV replication and cytokine induction. The levels of viral NS3 protein expression and viral progeny production were lower in cells cultured with PA-BSA than BSA (Figs. 1C and S2B) and this reduction could be rescued by serum supplement (Figs. 1D and S2C). The induction of IL-6 and TNF-α was higher in JEV-infected cells cultured with PA-BSA (Figs. 1E, 1F, S2D, S2E), even though viral replication was reduced under this condition. Interleukin 10 (IL-10), but not IL-4 and IL-13, was also induced in JEV-infected cells cultured with PA-BSA (S3 Fig). Furthermore, this cytokine induction depended on ROS generation, because of stronger ROS signals seen in JEV-infected cells cultured with PA-BSA than BSA (S4A Fig) and treatment with N-acetylcysteine (NAC), a free radical scavenger, reduced the levels of cytokine induction (Fig. 1E and 1F). Moreover, nuclear translocation of NFκB, an indicator of NFκB activation, was more prominent in JEV-infected cells cultured with PA-BSA than that with BSA (91% vs. 62%) (S4B Fig). Our data thus suggest that JEV-impaired LCFA β-oxidation can lead to ROS generation, NFκB activation and cytokine induction.

### JEV NS5 interacts with MTP subunits HADHα and HADHβ

**Fig 2:**
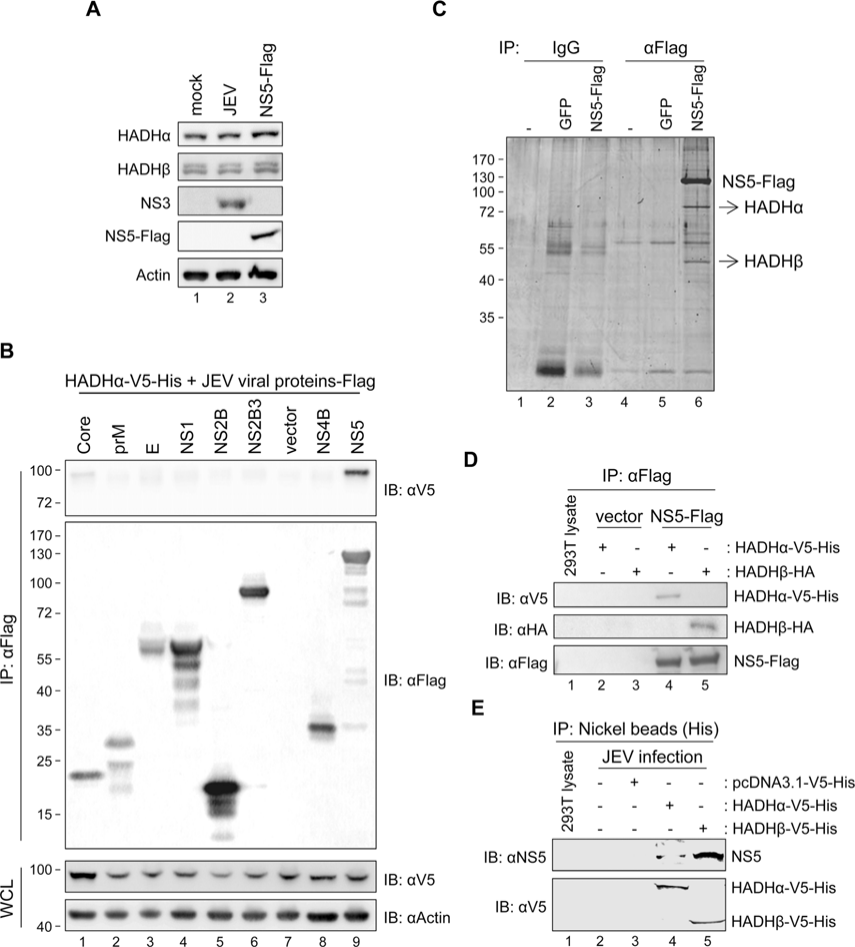
JEV NS5 interacts with mitochondrial trifunctional protein (MTP), the enzyme complex involved in LCFA β-oxidation. (A) Western blot analysis of protein levels of the indicated proteins in A549 cells with JEV infection (MOI = 10) or NS5-Flag overexpression. (B) Western blot analysis of V5-tag, Flag-tag, and actin in HEK293T cells co-transfected with HADHα-V5-His plus the indicated plasmids expressing Flag-tagged JEV viral proteins for 24 h, then immunoprecipitated with anti-Flag affinity gel. WCL, whole-cell lysates. (C) Immunoprecipitation (IP) analysis with control IgG or anti-Flag affinity gel in A549, GFP-A549 and NS5-Flag-A549 cells. The protein bands identified as HADHα and HADHβ are indicated by arrows. (D) IP with anti-Flag affinity gel and Western blot analysis with the indicated antibodies in HEK293T cells co-transfected with vector control or NS5-Flag plus HADHα-V5-His or HADHβ-HA for 24 h. (E) IP analysis with nickel beads and Western blot analysis with the indicated antibodies in HEK293T cells adsorbed with JEV for 3 h, then transfected with vector control, HADHα-V5-His or HADHβ-V5-His for 24 h.

**Fig 3:**
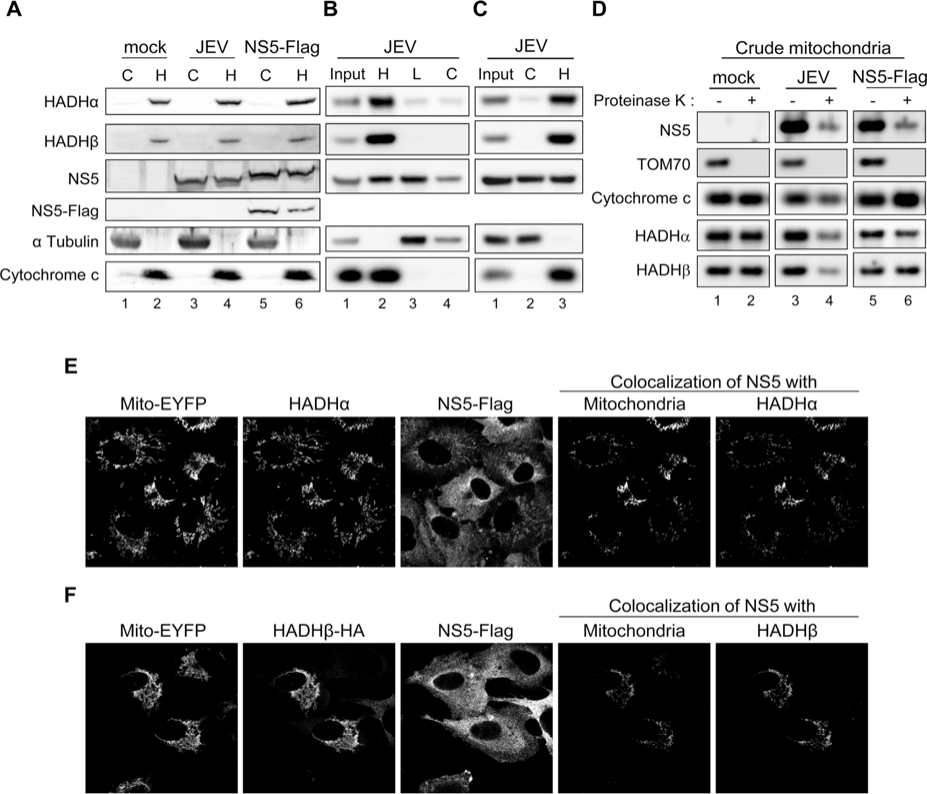
Subcellular localization of JEV NS5. (A-C) Cellular lysates of HEK293T cells infected with JEV (MOI = 5) or transfected with NS5-Flag for 24 h underwent Qproteome Mitochondria Isolation (A) or biochemical fractionation as outlined in S5B and S5C Fig, respectively (B and C). Western blot analysis of indicated proteins in cytosolic and crude mitochondrial fractions. C, cytosolic fraction; H, heavy membrane fraction/crude mitochondrial fraction; L, light microsomal membrane fraction. (D) The crude mitochondrial fraction isolated from HEK293T cells infected with JEV (MOI = 3) or transfected with NS5-Flag for 24 h was treated with Proteinase K (100 μg/ml) for 30 min on ice. The reactants were developed by Western blot analysis with antibodies against NS5 or the indicated mitochondrial proteins. (E) Confocal microscopy of pEYFP-Mito-NS5-A549 cells stained with anti-Flag plus Alexa Fluor 568 goat anti-rabbit and anti-HADHα plus Alexa Fluor 647 goat anti-mouse antibody. (F) Confocal microscopy of pEYFP-Mito-NS5-A549 cells transfected with HADHβ-HA for 24 h and stained with anti-Flag plus Alexa Fluor 568 goat anti-rabbit and anti-HA plus Alexa Fluor 647 goat anti-mouse antibody.

Three of the four enzymatic activities of LCFA β-oxidation are catalyzed by protein complex MTP [14], so we investigated whether JEV modulates LCFA β-oxidation by changing the expression and/or localization of MTP. The protein expression levels of the two subunits of MTP, HADHα and HADH, were similar between JEV- and mock-infected cells (Fig. 2A, lanes 1–2). Furthermore, by using a mitochondria isolation kit (S5A Fig), these two MTP proteins were detected in the heavy membrane fraction (H) containing mitochondria of mock- and JEV-infected cells (Fig. 3A, lanes 2 and 4). We then explored whether certain JEV proteins might interact with MTP by using immunoprecipitation (IP)–Western analysis. The plasmids expressing individual Flag-tagged JEV proteins were co-transfected with that of V5-tagged HADHα. In cells expressing HADHα-V5 plus NS5-Flag but not other viral proteins, anti-Flag affinity gel also brought down V5-tagged HADHα (Fig. 2B). Furthermore, endogenous HADHα and HADHβ were identified as NS5-interacting proteins by LC-MS/MS proteomic analysis of cellular proteins co-immunoprecipitated with Flag-tagged NS5 (Figs. 2C and S6). The interaction of NS5-Flag with HADHα-V5-His and HADHβ-HA was demonstrated by IP—Western analysis (Fig. 2D). The virus-expressed NS5 also interacted with HADHα and HADHβ, as demonstrated by IP—Western analysis of cellular lysates with JEV infection plus HADHα-V5-His or HADHβ-V5-His transfection (Fig. 2E).

JEV NS5 protein expressed by plasmid transfection or viral infection was detected in cytosolic (C) and mitochondria-containing heavy membrane (H) fractions (Fig. 3A, 3B and 3C) by using 3 different isolation protocols outlined in S5 Fig. To better understand the subcellular localization of JEV NS5, we performed Proteinase K resistance assay on the crude mitochondria isolated from HEK293 cells with JEV infection or JEV NS5-Flag overexpression. As shown in Fig. 3D, Proteinase K digested the mitochondrial outer membrane protein TOM70, whereas the proteins in intermembrane-space (Cytochrome c) and inner-membrane (HADHα and HADHβ) were protected. Importantly, some of the NS5 proteins were resistant to Proteinase K cleavage, suggesting enclosure of NS5 by membrane structure. JEV NS3 and E proteins were detected in the cytosolic and membrane-containing fractions (S5 Fig) as previously reported [38,39]. Furthermore, E but not NS3 protein was resistant to Proteinase K-mediated cleavage (S5C Fig), in accordance with the known locations for NS3 and E in cytosol and inside the endoplasmic reticulum (ER), respectively. Different fractionation patterns were noted between mock- and JEV-infected cells; for example, the ER protein calreticulin was mainly detected in the cytosolic/light microsomal membrane fractions of mock cells, but its location slightly shifted to the heavy membrane fraction (S5B Fig), probably due to the intracellular membrane rearrangements known to be caused by many positive-sense RNA viruses, including JEV [11]. Furthermore, co-localization of NS5 with mitochondria and with HADHα or HADHβ was detected by confocal microscopy (Fig. 3E and 3F). Similar to with JEV infection, NS5 protein expression did not change the expression level (Fig. 2A, lane 3) or cellular location (Fig. 3A, lanes 5–6) of HADHα and HADH. Thus, JEV NS5 can locate in a rearranged heavy membrane structure containing mitochondrial proteins and interact with subunits HADHα and HADHβ of the LCFA β-oxidation enzyme complex MTP.

### JEV NS5 is involved in impaired LCFA β-oxidation and cytokine induction

**Fig 4:**
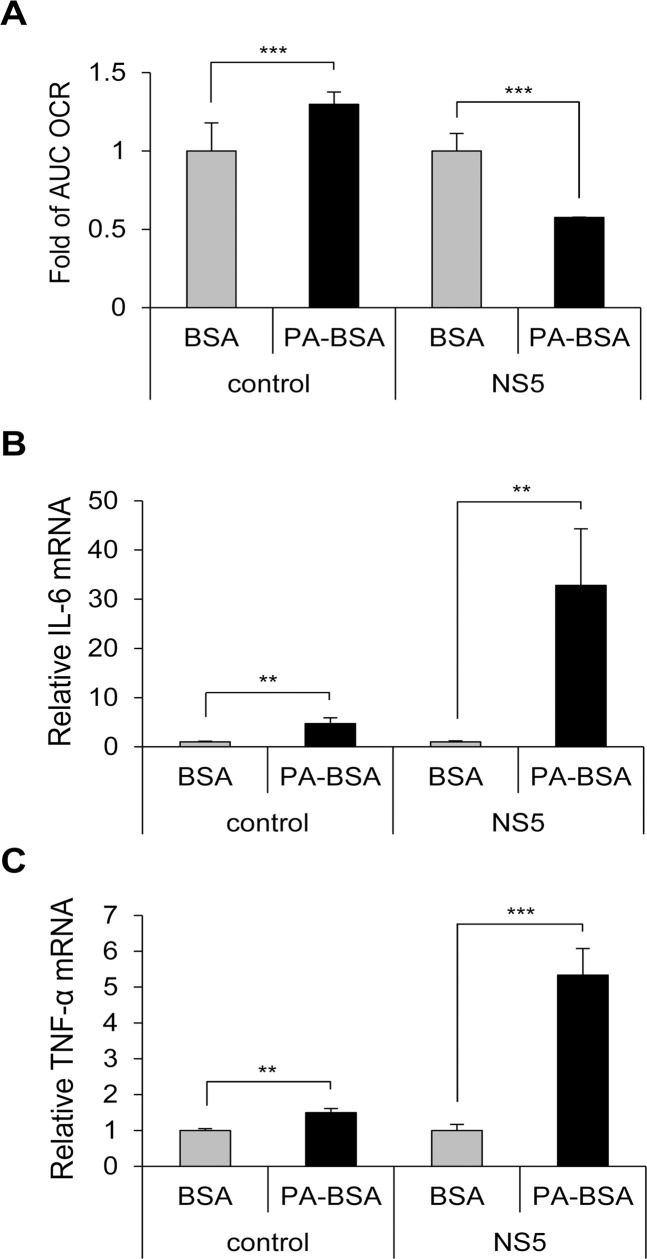
Impaired LCFA β-oxidation and cytokine induction in NS5-overexpressing cells. (A) AUC OCR for NS5-overexpressing and vector control-A549 cells incubated with serum-free medium for 1 h, then treated with PA-BSA or BSA control for 18 h (n = 2). (B and C) NS5-overexpressing and vector control-A549 cells were cultured with serum-free medium for 1 h, then incubated with PA-BSA or BSA for 24 h. RT-qPCR analysis of the relative mRNA levels of IL-6 (B) and TNF-α (C) (n = 3). Data are mean±SD. **P < 0.01, ***P < 0.001.

To test whether NS5 is involved in impaired LCFA β-oxidation, we measured the OCR of A549 cells with or without NS5 overexpression cultured with PA-BSA or BSA. The AUC OCR was significantly lower in NS5-overexpressing cells cultured with PA-BSA than BSA, whereas AUC OCR was higher in vector control cells cultured with PA-BSA than BSA (Fig. 4A). Levels of IL-6 and TNF-α were higher in NS5-overexpressing cells cultured with PA-BSA than BSA (Fig. 4B and 4C). Moreover, this cytokine induction phenomenon was JEV NS5-specific, since cells expressing other viral proteins such as JEV NS1, NS2A and DENV NS2B3 did not show TNF-α induction with PA-BSA treatment (S7 Fig).

### Binding of NS5 with MTP associated with its ability to block LCFA β-oxidation and induce cytokine production

**Fig 5:**
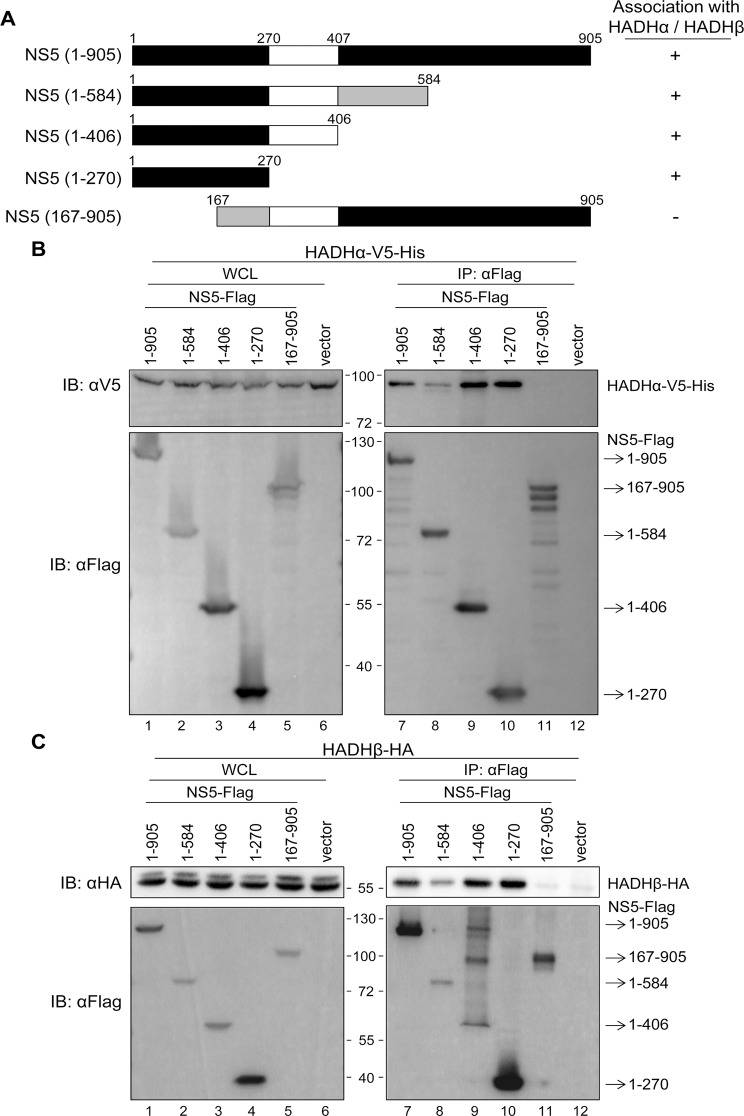
N-terminus of NS5 is essential for its interaction with MTP. (A) Schematic diagram and properties of full-length and truncated NS5 constructs. (B and C) IP—Western and Western blot analysis with anti-Flag affinity gel and the indicated antibodies for Flag-tag, V5-tag and HA-tag in HEK293T cells co-transfected with full-length and truncated NS5-Flag plus HADHα-V5-His (B) or HADHβ-HA (C) for 24 h.

**Fig 6:**
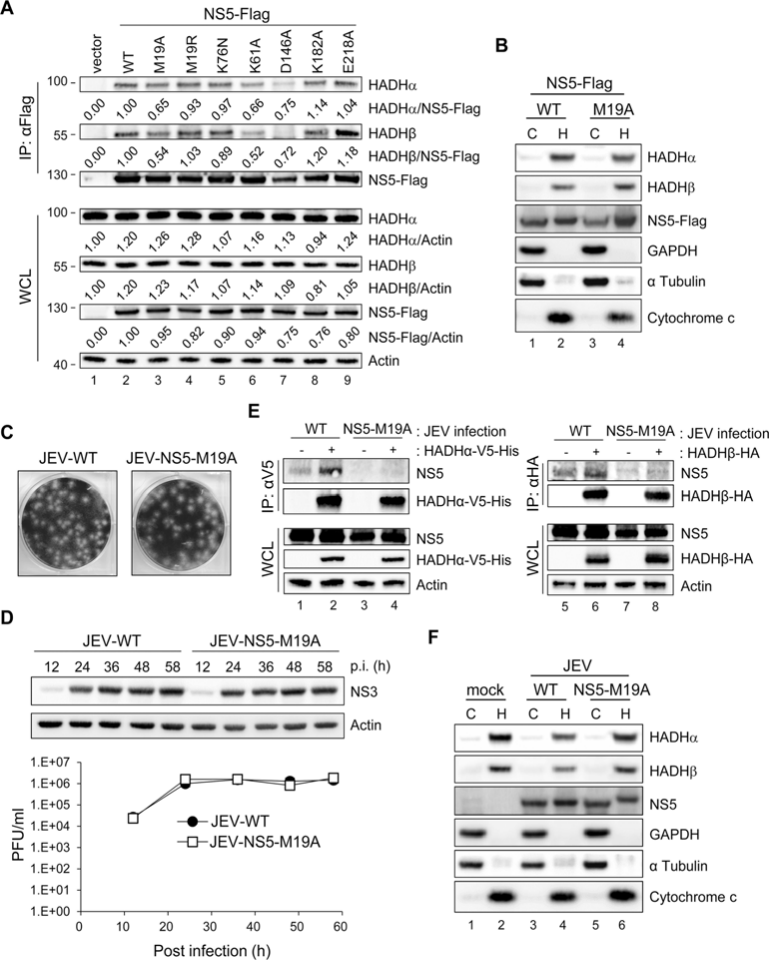
NS5 with mutation on residue 19 (M19A) showed reduced binding ability with MTP. (A) Western blot analysis of indicated proteins in HEK293T cells transfected with the plasmids expressing wild type (WT)-, mutated-NS5-Flag or vector control for 24 h after immunoprecipitation with anti-Flag affinity gel. Band densities were quantified by use of MetaMorph (Molecular Devices). (B and F) HEK293T cells transfected with NS5-Flag or NS5-M19A-Flag (B) or infected with wild-type JEV (JEV-WT) or JEV-NS5-M19A (MOI = 5) (F) for 24 h underwent Qproteome Mitochondria Isolation. Western blot analysis of indicated proteins in mitochondrial and cytosolic fractions. C, cytosolic fraction; H, heavy membrane fraction/crude mitochondrial fraction. (C and D) JEV-NS5-M19A mutant virus was generated by using a JEV infectious clone. (C) Plaque morphology of wild type JEV (JEV-WT) and JEV-NS5-M19A mutant in BHK-21 cells. (D) A549 cells were infected with JEV-WT or JEV-NS5-M19A (MOI = 0.1) for the indicated times. Western blot analysis of protein levels of NS3 and actin. Plaque-forming assay of virus titration in culture supernatants (n = 3). Data are mean±SD. (E) IP analysis with V5 or HA affinity gel and Western blot analysis with the indicated antibodies in HEK293T cells adsorbed with JEV for 1 h, then transfected with HADHα-V5-His or HADHβ-HA for 24 h.

To identify the region of NS5 interacting with HADHα or HADH, we co-expressed Flag-tagged full-length or a series of truncated NS5 used previously [7] (Fig. 5A), with V5-tagged HADHα or HA-tagged HADHβ. Anti-Flag affinity gel co-immunoprecipitated HADHα and HADHβ with the NS5 proteins containing N-terminal 1–270 residues but not with the N-terminal—deleted NS5 (167–905) (Fig. 5B and 5C). To identify the crucial amino acids of NS5 (1–270) participating in this interaction, we created NS5 mutants by random mutagenesis and screened for their ability to bind with HADHα or HADHβ by IP—Western analysis. The NS5 mutant with residue 19 changed from methionine to alanine (M19A) showed reduced binding with endogenous HADHα and HADH (Fig. 6A), despite the cellular distribution of NS5-WT and NS5-M19A was similar (Fig. 6B). To verify whether the MTase activity located at the N-terminus of NS5 is involved in the interaction with MTP, we site-specifically mutated the enzyme catalytic tetrad KDKE motif [40] by creating the K61A, D146A, K182A and E218A mutants of JEV NS5. NS5-K61A and-D146A, but not-K182A and-E218A mutants, showed reduced binding with MTP (Fig. 6A), which suggests that the MTase enzyme activity per se is not essential for this protein—protein interaction.

To test whether NS5 association with MTP is involved in impaired LCFA β-oxidation, we compared the OCR of A549 cells with wild-type NS5 (NS5-WT) or NS5-M19A overexpression. Higher AUC OCR was noted in PA-BSA-treated NS5-M19A cells (S8A Fig), indicating that NS5-M19A was less able to block LCFA β-oxidation than NS5-WT. Consistently, lower IL-6 and TNF-α induction was seen in cells expressing NS5–M19A (S8B and S8C Fig). Furthermore, IL-6 protein level was higher in NS5-overexpressing cells when compared to GFP control and NS5-M19A mutant (S8D Fig).

We then created recombinant JEV with NS5 mutation by using a JEV infectious clone [41]. Since NS5-K61A mutation hampers JEV replication [42], we selected NS5-M19A and-D146A for recombinant JEV generation. JEV with NS5-M19A, but not-D146A mutation was recovered, likely because the D146A mutation will abolish its MTase activity and lose viral replication ability as reported for WNV [43]. JEV-NS5-M19A was infectious and produced similar plaque morphology as with wild-type JEV (JEV-WT) in BHK-21 cells (Fig. 6C). The viral NS3 protein expression and viral progeny production of JEV-WT and JEV-NS5-M19A were similar in A549 cells (Fig. 6D). However, the binding of NS5-M19A to HADHα-V5-His and HADHβ-HA was lower than that of NS5-WT in the context of virus infection (Fig. 6E), despite their cellular localization was similar (Fig. 6F).

**Fig 7:**
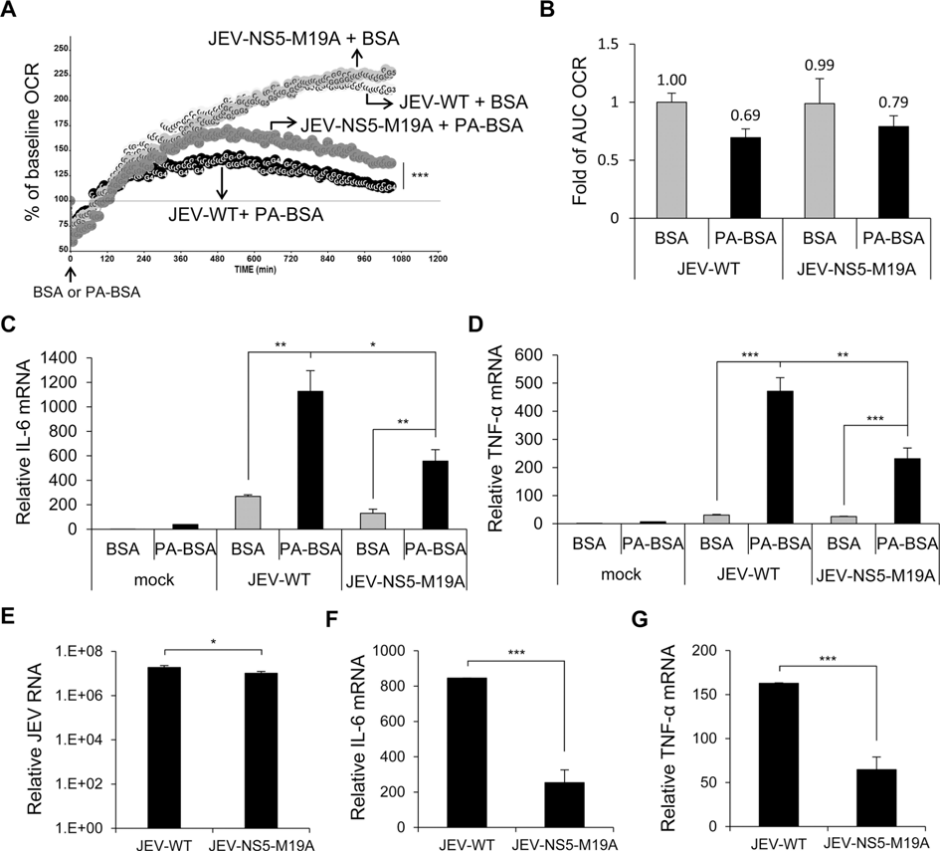
The recombinant JEV with NS5-M19A mutation is less able to block LCFA β-oxidation and induces less cytokine expression. (A and B) A549 cells infected with JEV-WT or JEV-NS5-M19A (MOI = 10) for 5 h were changed to serum-free medium for 1 h, then incubated with PA-BSA or BSA control. (A) Real-time OCR was measured from 6 to 24 h post-infection. The OCR before PA-BSA or BSA treatment was set to 100%. (B) The AUC OCR with PA-BSA and BSA (n = 3). (C and D) A549 cells infected with the indicated JEV (MOI = 10) for 5 h were incubated with serum-free medium for 1 h before treatment with PA-BSA or BSA for 18 h. RT-qPCR analysis of relative mRNA levels of IL-6 (C) and TNF-α (D) (n = 3). (E-G) A549 cells were infected with JEV-WT or JEV-NS5-M19A (MOI = 10) for 24 h in serum (10% FBS)-containing medium. RT-qPCR analysis of relative mRNA levels of JEV RNA (E), IL-6 (F) and TNF-α (G) (n = 3). Data are mean±SD.*P < 0.05, **P < 0.01 and ***P < 0.001.

The OCR and AUC OCR were higher in PA-BSA—treated A549 cells infected with JEV-NS5-M19A than JEV-WT, while with similar values with BSA treatment (Fig. 7A and 7B). Thus, JEV-NS5-M19A was less able to block LCFA β-oxidation and induced lower levels of IL-6 and TNF-α than JEV-WT (Fig. 7C and 7D). Furthermore, as compared with JEV-WT infection, even with serum-containing medium, JEV-NS5-M19A infection triggered significantly lower levels of IL-6 and TNF-α while producing slightly less viral RNA (Fig. 7E, 7F and 7G). Thus, M19 of NS5 is involved in its interaction with MTP and affects the ability of JEV to impair LCFA β-oxidation and induce cytokine expression.

### Reduced interaction between NS5 and MTP attenuates JEV virulence

**Fig 8:**
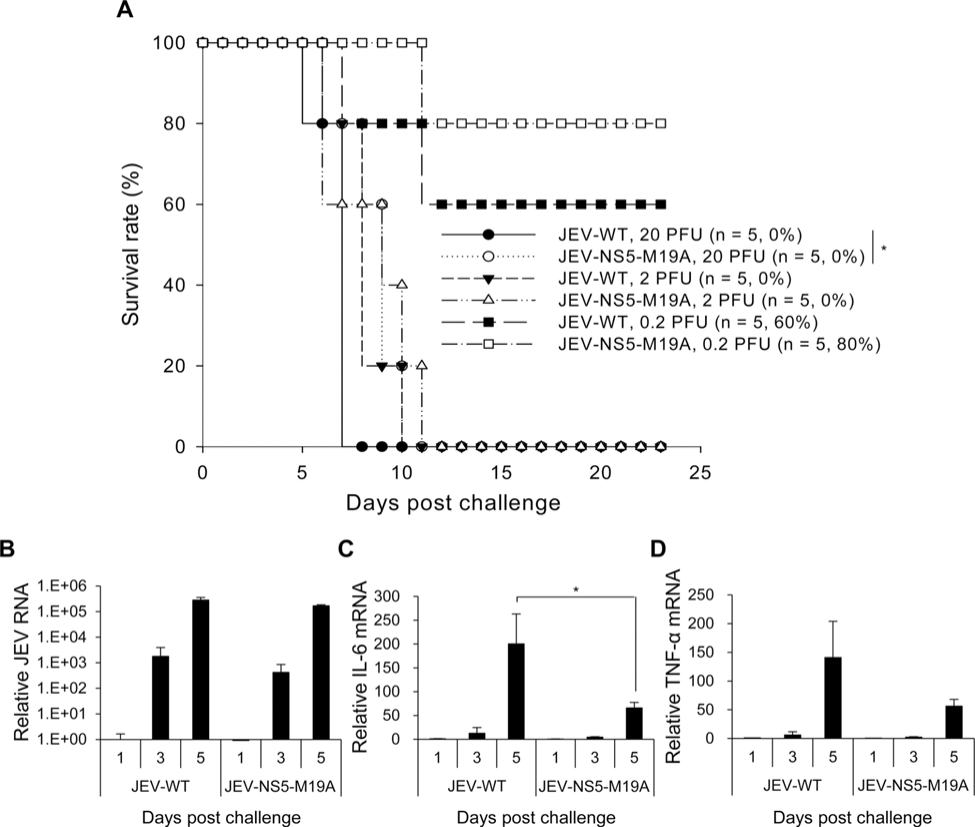
Reduced neurovirulence of NS5-M19A—mutated JEV in challenged mice. (A) Survival in C57BL/6 mice infected with 0.2, 2 or 20 plaque-forming units (PFU) of JEV-WT or JEV-NS5-M19A by an intracerebral (i.c.) injection. The animal number (*n*) and survival rate for each group are shown. (B-D) RT-qPCR of relative JEV RNA (B), IL-6 (C), and TNF-α (D) mRNA levels in brain tissues of mice inoculated with JEV-WT or JEV-NS5-M19A (20 PFU) (n = 3). Data are mean±SD.*P < 0.05.

To investigate the impact of LCFA β-oxidation on JEV infection in vivo, we compared the neurovirulence of JEV-WT and JEV-NS5-M19A in mice with intracerebral (i.c.) virus injection. All mice died with injection of 20 or 2 plaque-forming units (PFU) of JEV-WT or JEV-NS5-M19A, whereas 80% and 60% of mice survived from challenge with 0.2 PFU of JEV-NS5-M19A and JEV-WT, respectively (Fig. 8A). The 50% lethal dosage (LD_50_) was calculated as 4 x 10^-1^ and 9.48 x 10^-1^ PFU for JEV-WT and JEV-NS5-M19A, respectively, for a 2.37-fold increase for JEV-NS5-M19A. The levels of viral RNA replication were similar in the brains of mice infected with JEV-WT or JEV-NS5–M19A (Fig. 8B). However, IL-6 and TNF-α induction was higher in brains of mice infected with JEV-WT than JEV-NS5–M19A (Fig. 8C and 8D).

**Fig 9:**
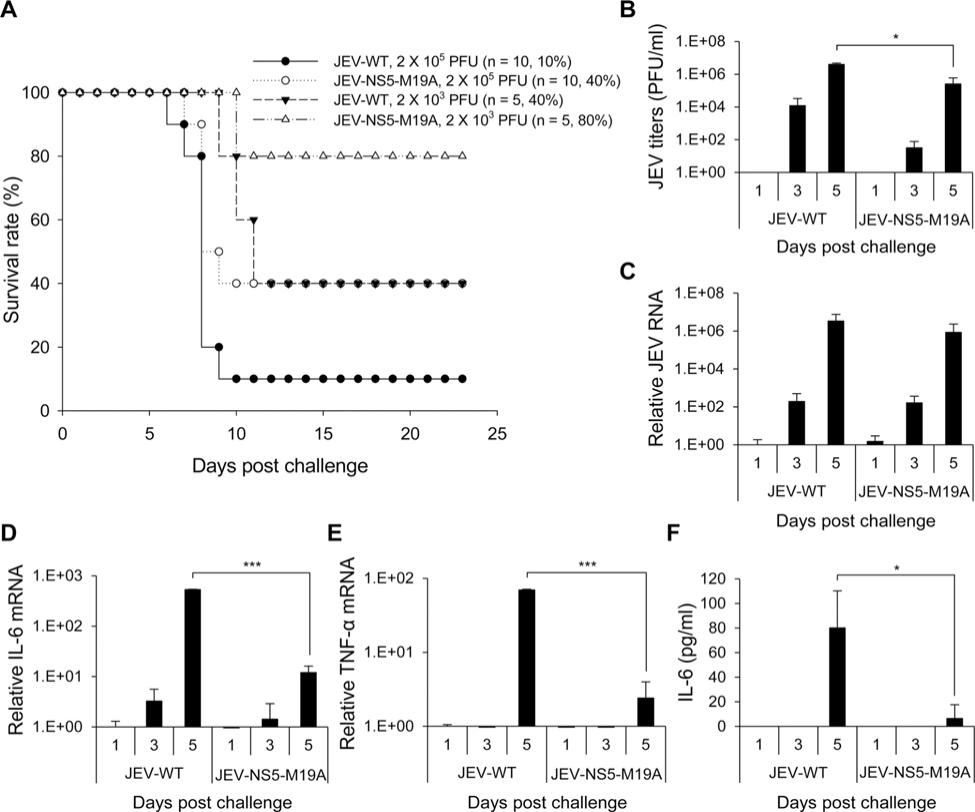
Reduced neuroinvasiveness of NS5-M19A—mutated JEV in challenged mice. (A) Survival in C57BL/6 mice infected with 10^3^ or 10^5^ PFU JEV-WT or JEV-NS5-M19A by an intraperitoneal (i.p.) plus i.c. route. The animal number (*n*) and survival rate for each group are shown. (B) Plaque-forming assay of virus titers in brain tissues of mice inoculated with JEV-WT or JEV-NS5-M19A (10^5^ PFU) (n = 3). (C-E) RT-qPCR of relative JEV RNA (C), IL-6 (D), and TNF-α (E) mRNA levels in brain tissues (n = 3). (F) ELISA of IL-6 protein levels in the sera samples (n = 3). Data are mean±SD.*P < 0.05, ***P < 0.001.

The difference between these two viruses was more obvious on challenge with an intraperitoneal (i.p.) injection plus i.c. puncture with PBS (i.p. plus i.c. route). The LD_50_ for JEV-WT and JEV-NS5-M19A was 2 x 10^3^ and 1.38 x 10^4^ PFU, respectively, for a 6.92-fold increase for the NS5-M19A mutated JEV (Fig. 9A). The levels of JEV titers and viral RNA were higher in mouse brains inoculated with JEV-WT than JEV-NS5–M19A (Fig. 9B and 9C). Furthermore, IL-6 and TNF-α gene induction was higher in mouse brains challenged with JEV-WT than JEV-NS5–M19A (Fig. 9D and 9E) and IL-6 protein could be detected in the sera of mice with JEV-WT infection (Fig. 9F). Thus, JEV NS5 can bind with MTP and hinder its ability to catalyze LCFA β-oxidation, which then induces cytokine production and contributes to viral pathogenesis.

## Discussion

Flaviviral NS5 contains 2 enzymatic domains: RdRP on its C-terminus required for viral RNA replication and MTase on its N-terminus needed for viral RNA stability and efficient translation [4,5]. The 2'-O methylation on the viral RNA 5' cap catalyzed by NS5 MTase contributes to escape from the IFIT-mediated host antiviral response for WNV and JEV [44,45]. Several flaviviral NS5 proteins such as those from JEV, WNV, TBEV and DENV have been identified as IFN signaling antagonists [7–10] by targeting STAT2 and not-yet-identified mechanisms. Here, we discover a new function of flaviviral NS5: JEV NS5 interacts with MTP, an enzyme complex involved in LCFA β-oxidation and interferes with the catabolism of LCFA. The accumulated LCFA triggers oxidative stress, activates NFκB, induces pro-inflammatory cytokine production and contributes to JEV pathogenesis. Thus, besides being the enzyme involved in virus replication, flaviviral NS5 also functions as an immune modulator by affecting the host immune system such as IFN signaling and cytokine production. Furthermore, these two immunomodulation functions of NS5 may not be mediated by the same molecular mechanism, since JEV-WT and JEV-NS5-M19A show different degree of LCFA β-oxidation impairment, but both can trigger IFN production and block IFN signaling (S9 Fig).

Different cellular distribution has been reported for flaviviral NS5 proteins. For example, the NS5 proteins of DENV-2 and DENV-3 [46,47] mainly locate in the nuclei, but those of JEV [7,48,49], WNV [8,46,50], DENV-1 and DENV-4 [47] are in the cytoplasm. By using fractionation and confocal microscopy assays, JEV NS5 was detected in the cytosolic fraction and membrane-containing fractions including mitochondria (Figs. 3 and S5). Although no conventional mitochondria targeting sequence (MTS) was predicted, JEV NS5 shows ~1/3 in probability of translocation to mitochondria (Mitoprot score: 0.3351) by Mitoprot software (http://ihg.gsf.de/ihg/mitoprot.html) [51,52]. We suspect that the nonconventional mitochondria import pathways such as that used by microtubule-associated protein 4 (MAP4) [53] and human apurinic/apyrimidinic endonuclease [54] might be adapted by JEV NS5. Another possibility is that JEV NS5 may enter mitochondria with the help of other cellular proteins such as Hdj2, which is known to regulate mitochondrial protein import [55] and has been reported to interact with JEV NS5 [56]. Furthermore, the NS5 proteins in the crude mitochondrial fractions migrated slightly slower than the ones in cytosolic fractions (Fig. 3A). Thus, JEV NS5 might gain access to mitochondria through certain protein modifications such as the phosphorylation-mediated mitochondrial translocation of cytosolic proteins [57] reported for MAP4 [53] and Parkin [58]. However, mitochondrial translocation of NS5 does not guarantee its interaction with MTP, since the N-terminal—deleted NS5 (167–905) and M19A-mutated NS5 show reduced binding with MTP (Figs. 5 and 6) but could still be detected in mitochondria (Figs. 6 and S10A).

The recombinant JEV-NS5-M19A mutant and JEV-WT replicated to similar levels, but JEV-NS5-M19A was less able to block LCFA β-oxidation and triggered lower cytokine levels than the wild type in cultured cells (Figs. 6 and 7). Furthermore, JEV-NS5-M19A exhibited attenuated neurovirulence and neuroinvasiveness as compared with JEV-WT in challenged mice (Figs. 8 and 9). According to the crystal structure of JEV NS5 [59], M19 residue is located on a linker between two helix structures (S10B Fig). Since linker peptide mutants may affect protein folding and lead to conformational changes [60,61], we suggest that structural integrity of the linker with residue M19 on NS5 may be essential for maintaining its functional interaction with MTP.

Similar to our findings with JEV, infection with HCV and human cytomegalovirus (HCMV) impairs fatty acid β-oxidation [25,62], whereas DENV infection increases fatty acid β-oxidation [63]. We also noted that DENV-2 infection was less able to block β-oxidation than JEV infection (S11A Fig). DENV-2 NS5 mainly located in cell nuclei and did not interact with MTP (S11B and S11C Fig). Furthermore, inhibition of β-oxidation by etomoxir reduced DENV replication [63] but has no effect on JEV replication (S12 Fig). Thus, NS5 proteins of JEV but not that of DENV-2 interact with cellular MTP and these two viruses interplay with cellular fatty acid β-oxidation in different ways.

Viruses use various mechanisms, such as by affecting gene expression and protein—protein interaction, to regulate fatty acid β-oxidation. Genes including HADHα and peroxisome proliferator-activated receptor α, a transcription factor required for the expression of genes involved in fatty acids metabolism, are downregulated in patients with HCV cirrhosis and hepatocellular carcinoma [26]. HCV core protein can induce various alterations in lipid metabolism by increasing the expression of genes involved in lipogenesis and decreasing that of genes involved in β-oxidation and secretion of fatty acids [25,64]. For HCMV infection, a cellular IFN-induced protein named Viperin is translocated into mitochondria to interact with MTP and inhibit fatty acid β-oxidation [62,65]. Since JEV infection causes protein degradation of Viperin [66], Viperin redistributed to mitochondria may not be adapted by JEV to block MTP. AMP-activated kinase (AMPK) plays a role in cellular energy homeostasis; activation of AMPK can inhibit fatty acid synthesis and restrict infection of several RNA viruses [67]. We also addressed whether AMPK is involved in JEV infection by treating cells with an AMPK activator A769662, which showed no effect on JEV replication in cells with or without palmitate pretreatment (S13B and S13C Fig). Thus, different from Kunjin virus [67], AMPK may not be involved in JEV replication and may not contribute to impaired β-oxidation in JEV-infected cells.

Under the well-fed condition, glucose is the major substrate for ATP production, but when glucose level is low or with excess fatty acid content, fatty acids will become the alternative source for energy production [36,37]. JEV infection consumes ATP [68], so ATP levels were lower in JEV-infected cells as compared to mock (S14 Fig). Because of LCFA β-oxidation impairment by JEV, further reduction of ATP was seen in cells cultured with PA-BSA (S14 Fig). Normally, glucose is mainly broken down by oxidative phosphorylation in mitochondria, but under hypoxia and stress conditions such as virus infection [69], glycolysis occurring in cytoplasm will dominate [70,71]. For example, HCV and HCMV infection induces glycolysis [72,73] and the activity of some glycolysis enzymes is increased during JEV infection [68,74]. Glycolysis produces lactate and causes acidification of the extracellular space, called lactic acidosis [75], seen in patients with LCFA β-oxidation deficiency [76,77] and Japanese encephalitis (JE) [78]. Thus, the high lactate secretion in patients with JE might be a metabolic symptom due to impaired LCFA β-oxidation during JEV infection.

Fatty acids can generate intracellular ROS via several mechanisms [79] and fatty acid metabolism has been implicated in viral pathogenesis. For example, the expression of pro-inflammatory cytokines IL-6 and TNF-α was higher in hepatitis B virus X-protein—transgenic mice fed a high-fat rather than normal diet [80]. Furthermore, a non-neurotropic influenza A virus replicated to increased levels in mice lacking carnitine transporter OCTN2, a gene required for LCFA β-oxidation, and resulted in increased brain vascular permeability and encephalopathy [29]. Thus, disordered mitochondrial β-oxidation increases the risk of brain damage caused by influenza A virus infection. JEV is a neurotropic virus [5] that causes encephalitis by attracting immune cells across the blood brain barrier to induce the inflammatory response and brain pathology [30–32]. When fatty acid β-oxidation is impaired, the accumulation of LCFA elicits protein oxidative damage and decreases antioxidants in the cerebral cortex [18]. Thus, impaired LCFA β-oxidation may facilitate membrane proliferation and rearrangement in JEV-infected cells, but then likely contributes to JE-associated brain damage. Our finding that JEV NS5 associates with MTP and can inhibit fatty acid β-oxidation may shed new light on JEV-triggered pathogenesis and provide a novel target for future drug development.

## Materials and Methods

### Cell lines, viruses and chemicals

Human lung epithelial carcinoma A549 cells (ATCC, CCL-185) were cultured in F-12 medium (Invitrogen, Grand Island, New York, USA) containing 10% fetal bovine serum (FBS). Human neuroblastoma SK-N-SH cells (ATCC, HTB-11) were grown in Minimum Essential Media (Invitrogen) containing 10% FBS. Human embryonic kidney 293T cells (HEK293T; ATCC, CRL-11268) were cultured in Dulbecco’s modified Eagle’s medium (Invitrogen) containing 10% FBS. Baby hamster kidney BHK-21 cells (ATCC, CCL-10) were grown in RPMI 1640 medium (Invitrogen) containing 5% FBS. JEV strain RP-9 [81] (GenBank accession no. AF014161) was propagated in the C6/36 mosquito cell line grown in RPMI 1640 medium supplemented with 5% FBS.

The JEV-NS5-M19A mutant was generated by single-primer mutagenesis [82] with the primer 5΄-GAAGGAAAAACTAAATGCCGCGAGCAGAGAAGAGTTTTTTAAATACCG-3΄ (mutated sequence underlined) with a JEV infectious clone as described [41]. For viral infection, cells were adsorbed with virus at the indicated multiplicity of infection (MOI) for 2 h at 37°C, then unbound virus was removed by a gentle wash with HBSS (Invitrogen). At the indicated times post-infection, culture supernatants were sequentially diluted for plaque-forming assays on BHK-21 cells as described [41]. To establish JEV NS5-overexpressing cells with EYFP-tagged mitochondria, pTY-EF-NS5-Flag cells [7] were transduced with EYFP-Mito-expressing lentivirus for 24 h, then selected with 5 μg/ml puromycin for 72 h.

N-acetylcysteine (NAC) (A7250), Proteinase K (P2308), recombinant human IFN-αA/D (I4401), and Etomoxir (E1905) were from Sigma (St. Louis, MO, USA). A769662 (sc-203790) was from Santa Cruz Biotechnology (Dallas, Texas, USA).

### Cytotoxicity test

Cytotoxicity was assessed by use of the Cytotoxicity Detection Kit (LDH) (Roche, Basel, Switzerland). Cell viability was determined by using AlamarBlue (Invitrogen) cell viability assay and trypan blue exclusion assay (Gibco, Grand Island, NY, USA). Briefly, A549 cells were incubated with the indicated concentration (0–400 μM) of PA-BSA for 24 h. Cell-free supernatants were collected and used in LDH assay as instructed by the manufacturer. The viable cells stained with AlamarBlue were determined by measurement of spectrophotometric absorbance with a microplate reader. The cells were mixed with an equal volume of trypan blue then survival cell numbers were determined using an automated cell counter (Countess; Invitrogen).

### Measurement of LCFA β-oxidation

Oxygen consumption rate (OCR) in A549 and HTB11 cells was measured in serum-free F-12 medium containing 0.5 mM L-carnitine (C0158; Sigma), an essential addition to transport palmitate into mitochondria. Sodium palmitate (P9767; Sigma) was conjugated with fatty acid free bovine serum albumin (BSA) (A7030; Sigma) (PA-BSA) at a 6:1 molar ratio by a protocol from Seahorse Bioscience (North Billerica, MA, USA). Briefly, sodium palmitate was solubilized in 150 mM NaCl by heating up to 70°C. BSA was dissolved in 150 mM NaCl and warmed up to 37°C with continuous stirring. Solubilized palmitate was added to BSA at 37°C with continuous stirring. Then, the conjugated palmitate—BSA (PA-BSA) was aliquoted and stored at -20°C for assessing β-oxidation of long-chain fatty acid [35]. After the injection of PA-BSA or BSA, OCR values were real-time recorded every 8 min from 6 to 24 h post infection with use of an XF24 analyzer (Seahorse Bioscience) and the area under the curve (AUC) OCR was calculated.

### Western blot analysis

Cells were lysed with RIPA buffer (10 mM Tris, pH 7.5, 5 mM EDTA, 150 mM NaCl, 0.1% SDS, 1% TritonX-100, 1% sodium deoxycholate) containing a cocktail of protease inhibitors (Roche). Equivalent amounts of proteins determined by the DC Protein Assay Kit (Bio-Rad, Hercules, CA, USA) were separated by SDS-PAGE and transferred to a nitrocellulose membrane (Hybond-C Super; Amersham, Buckinghamshire, UK). Nonspecific antibody binding sites were blocked with skim milk in phosphate-buffered saline (PBS) with 0.1% Tween 20 (PBST), then reacted with primary antibodies for HADHα (sc-82185), HADH (sc-55661 and sc-271496) and α Tubulin (sc-5546) from Santa Cruz Biotechnology; actin (NB600-501; Novus Biologicals, Littleton, CO, USA); GAPDH (GTX100118) and TOM70 (GTX85353) from GeneTex (Irvine, CA, USA); Cytochrome C (#556433; BD, Franklin Lakes, New Jersey, USA); Calreticulin (#2891), phospho-STAT1 (Tyr701) (#9171), STAT1 (#9172), phospho-AMPKα (Thr172) (#2535), and AMPKα (#2532) from Cell Signaling Technology(Danvers, MA, USA); GFP (#11814460001; Roche); V5-tag (V8012) and Flag-tag (F7425) from Sigma; or HA-tag (MMS-101R; Covance, Princeton, New Jersey, USA), and then incubated with an appropriate horseradish peroxidase-conjugated secondary antibody (Amersham). Signals were detected by enhanced chemiluminescence (Amersham).

### Quantitative RT-PCR (RT-qPCR)

Total cellular RNA was prepared with use of an RNeasy Mini Kit (Qiagen, Hilden, Germany) and cDNA was reverse-transcribed from 1 μg total RNA by use of the SuperScript III First-Strand Synthesis System (Invitrogen). qPCR involved use of TaqMan Universal PCR Master Mix (Invitrogen) with commercial probes for IL-6 (Hs00985639 and Mm00446190), TNF-α (Hs01113624 and Mm00443260), IL-10 (Hs00961622), IL-4 (Hs00174122), IL-13 (Hs00174379), IFN-β (Hs01077958) and GAPDH (Hs02758991 and Mm99999915) (Applied Biosystems, Foster City, CA, USA) as well as primers for JEV viral RNA (5΄-AGAACGGAAGATAACCATGACTAAA-3΄and 5΄-CCGCGTTTCAGCATATTGAT-3΄). The relative expression of genes was assessed by the comparative threshold cycle method and normalized to that of GAPDH.

### Measurement of intracellular ROS

Cells were stained with 50 μM 2’,7’-dichlorofluorescin diacetate (DCFH-DA) (OxiSelec Intracellular ROS Assay Kit; Cell Biolabs, San Diego, CA, USA) for 30 min and examined under an inverted fluorescent microscope.

### Immunofluorescence analysis

Cells were fixed with 4% formaldehyde in PBS for 20 min at room temperature, then washed twice with PBS. Cells were permeabilized in PBS containing 0.2 or 0.5% Triton X-100 for 5 min and blocked with skim milk in PBS or 3% BSA in Tris-buffered saline (TBS), then incubated with primary antibodies for NFκB p65 (sc-372; Santa Cruz), HADHα (sc-374497; Santa Cruz), Flag-tag (F7425; Sigma), or HA-tag (MMS-101R; Covance) diluted in TBS with 2% BSA overnight at room temperature before being washed with TBS, then with appropriate Alexa Fluor-conjugated secondary antibodies (Alexa Fluor 647 goat anti-mouse [A21236] or Alexa Fluor 568 goat anti-rabbit [A11036] from Invitrogen) for 1 h at room temperature. Cells were photographed under a fluorescence microscope or a Zeiss LSM510 Meta Confocal Microscope with a 100X objective. Co-localization was visualized by use of the ZEN 2011 (Zeiss, Oberkochen, Germany) co-localization module.

### Isolation of mitochondrial fraction and Proteinase K resistance assay

The Qproteome Mitochondria Isolation Kit (Qiagen) was used to isolate crude mitochondria from HEK293T cells according to the manufacturer’s instruction as outlined in S5A Fig. Two other biochemical approaches of cellular fractionation were also performed as previously described [83,84]. As outlined in S5B Fig, cells were washed once with cold PBS, scraped off from culture plate, and lysed in homogenization buffer [20 mM HEPES (pH 7.5), 70 mM sucrose and 220 mM mannitol] by 30 strokes in a Dounce homogenizer. The homogenate was centrifuged at 800 g for 5 min to precipitate the nuclei, and the resulting supernatant was further centrifuged at 10,000 g for 10 min (4°C) to precipitate the crude mitochondrial fraction. The resulting supernatant was further centrifuged at 100,000 g for 30 min (4°C) to precipitate light membrane organelles, and the final supernatant was used as the cytosolic fraction. Another biochemical method [84] was outlined in S5C Fig. Briefly, JEV-infected HEK293T cells were washed once with cold PBS, scraped off from culture plate, and lysed in mitochondria buffer [10 mM Tris/MOPS (pH 7.4), 0.1 mM EGTA/Tris (pH 7.4) and 250 mM sucrose] by 20 strokes in a Dounce homogenizer. Part of the homogenate was centrifuged at 16,200 g for 30 min, and the resulting supernatant was used as cytosolic fraction. The rest homogenate was centrifuged at 600 g for 5 min to precipitate the nuclei and unbroken cells, and the resulting supernatant was further centrifuged at 7,000 g for 10 min (4°C). Then the resulting pellet was resuspended and centrifuged again at 10,000 g for 10 min (4°C) to precipitate the crude mitochondrial fraction. Isolated mitochondrial fractions were lysed and examined by Western blot analysis.

The Proteinase K resistance assay was performed as previously described [84]. Briefly, the crude mitochondria pellet was washed once with mitochondria buffer. Then, the pellet resuspended in mitochondria buffer was treated with Proteinase K on ice for 30 min. After adding 2 mM phenylmethylsulfonyl fluoride (PMSF) to quench the protease reaction, samples were centrifuged at 15,000 g for 10 min (4°C). The resulting pellet was washed with mitochondria buffer plus 1 mM PMSF and centrifuged again. The reactants were subjected to Western blot analysis with the indicated antibodies.

### Immunoprecipitation (IP)–Western analysis

HEK293T cells were transfected with the indicated plasmids by use of Lipofectamine 2000 (Invitrogen). After 24 h, cells were lysed with IP lysis buffer (50 mM Tris-HCl, 150 mM NaCl, 1 mM EDTA, 1% Triton X-100, pH 7.4) containing a cocktail of protease inhibitors (Roche). Cell lysates were immunoprecipitated with anti-Flag M2 (A2220),-HA (E6779),-V5 affinity gel (A7345) or Nickel beads (P6611; Sigma) overnight at 4°C. Proteins were eluted by sample buffer and examined by Western blot analysis with the indicated antibodies.

### Identification of NS5-associated proteins

Cell lysates of A549, GFP-A549 and NS5-Flag-A549 cells were immunoprecipitated with control IgG or anti-Flag affinity gel. Proteins in the immune complexes were separated by SDS-PAGE and visualized by staining with SYPRO Ruby (Invitrogen). The extra protein bands, which bound to NS5-Flag but not the control, were excised for in-gel trypsin digestion and analyzed by LC-MS/MS.

### Random mutagenesis

We created NS5 mutants by random mutagenesis with mutagenic dNTP analogs with the JBS dNTP-Mutagenesis Kit (PP-101; Jena Bioscience, Jena, Germany). The technique involves incorporation of the dNTP analogs 8-oxo-dGTP and dPTP, which induce base mispairing upon DNA amplification.

### Ethics statement

Animal studies were conducted according to the guidelines outlined by Council of Agriculture, Executive Yuan, Republic of China. The animal protocol was approved by the Academia Sinica Institutional Animal Care and Utilization Committee (Protocol ID 11-11-245). All surgery was performed under sodium pentobarbital anesthesia and every effort was made to minimize suffering.

### JEV virulence test in mice

Groups of 4-week-old C57BL/6 mice were challenged intracerebrally (i.c.) with 30 μl JEV-WT or JEV-NS5-M19A for neurovirulence testing or intraperitoneally (i.p.) with 500 μl JEV-WT or JEV-NS5-M19A and i.c. injected with 30 μl PBS (i.p. plus i.c.) to damage the blood-brain-barrier as previously described [85] for neuroinvasiveness testing [86].

### ELISA

A human IL-6 ELISA kit (BMS213INST; eBioscience, San Diego, CA, USA) was used to detect IL-6 secretion in PA-BSA treated A549 cells. A mouse IL-6 ELISA kit (EM2IL6; Thermo Fisher Scientific, Waltham, MA, USA) was used to detect IL-6 secretion in mouse sera samples.

### Measurement of ATP levels

Cells in 96-well plates were mixed and incubated with an equal volume of CellTiter-Glo Reagent (G7572; Promega, Fitchburg, WI, USA) for 12 min, and then ATP levels were determined by measurement of luminescent signal.

### Statistical analysis

Data are shown as mean±SD. The data for the AUC OCR was compared by ANOVA and post-hoc Tukey test with use of Prism 4 (GraphPad; La Jolla, CA, USA). The two-tailed Student *t* test was used for comparisons between 2 groups. The *p* values of survival curves were analyzed by the log-rank test using SigmaPlot 10 (Systat Software; San Jose, CA, USA). P < 0.05 was considered statistically significant.

## Supporting Information

S1 Fig PA-BSA cytotoxicity test. A549 cells were treated with solvent or PA-BSA (up to 400 μM) for 24 h. LDH (A), AlamarBlue (B), and trypan blue exclusion assays (n = 3) (C) were performed to determine cytotoxicity and cell viability. Data are mean±SD. *P < 0.05 and **P < 0.01.
(TIF)

S2 Fig Impaired long-chain fatty acid (LCFA) β-oxidation leads to inflammatory cytokine induction in JEV-infected neuroblastoma cells. (A) HTB11 cells infected with JEV (MOI = 10) for 5 h were replenished with serum-free medium for 1 h, then incubated with PA-BSA or BSA control. AUC OCR measured from 6 to 24 hpi compared to that for mock cells treated with BSA (n = 2 or 3). (B and C) HTB11 cells infected with JEV (MOI = 5 and 0.1) for 5 h were changed to medium without serum (B) or with serum (10% FBS) (C) for 1 h. Plaque-forming assay of cells treated with PA-BSA or BSA for 18 h before virus titration in culture supernatants (n = 3). (D and E) HTB11 cells infected with JEV (MOI = 10) for 5 h were replenished with serum-free medium for 1 h, then cultured with PA-BSA or BSA control. RT-qPCR analysis of relative mRNA levels of interleukin 6 (IL-6) (D) and tumor necrosis factor α (TNF-α) (E) (n = 3). Data are mean±SD. *P < 0.05, **P < 0.01, ***P < 0.001 and ns, not significant.
(TIF)

S3 Fig Impaired LCFA β-oxidation leads to IL-10 but not IL-4 or IL-13 induction in JEV-infected cells. A549 cells infected with JEV (MOI = 10) for 5 h were replenished with serum-free medium for 1 h, then treated with PA-BSA or BSA control for 18 h. RT-qPCR analysis of the relative mRNA levels of IL-10, IL-4 and IL-13 (n = 3). Data are mean±SD. *P < 0.05, ***P < 0.001 and ns, not significant.
(TIF)

S4 Fig Impaired LCFA β-oxidation leads to ROS production and NFκB activation in JEV-infected cells. A549 cells infected with JEV (MOI = 10) for 5 h were changed to serum-free medium for 1 h, then treated with PA-BSA or BSA. Fluorescence microscopy of cells stained with DCFH-DA for ROS production represented by green fluorescence (A), or stained with anti-NFκB p65 (green) plus DAPI (blue) (B).
(TIF)

S5 Fig Fractionation of JEV-infected cellular lysate. (A) HEK293T cells infected with JEV (MOI = 5) for 24 h were fractionated into cytosolic, nuclei & cell debris, microsomal and crude mitochondria by using Qproteome Mitochondria Isolation Kit. (B and C) Cellular fractions from HEK293T cells infected with JEV (MOI = 3) for 24 h by using the outlined procedure. 10 μg protein per fraction was analyzed by Western blot analysis for the indicated proteins. (C) The mitochondrial fraction isolated from JEV-infected HEK293T cells was treated with or without Proteinase K (100 μg/ml) for 30 min on ice. The reactants were developed by Western blot analysis with antibodies against NS3 and E. C, cytosolic fraction; L, light microsomal membrane fraction; H, heavy membrane fraction/crude mitochondrial fraction.
(TIF)

S6 Fig LC-MS/MS identification of the 83- and 51.3-kDa proteins. After LC-MS/MS analysis, 83-kDa protein band peptide sequences were matched to HADHα and 51.3-kDa protein band peptide sequences were matched to HADHβ shown in bold and underlined.
(TIF)

S7 Fig Impaired LCFA β-oxidation leads to cytokine induction in JEV NS5-overexpressing cells. A549 cells with JEV NS5, NS1, NS2A, DENV-2 NS2B3, or GFP control overexpression were cultured with serum-free medium for 1 h, then incubated with medium containing PA-BSA or BSA for 24 h. RT-qPCR analysis of the relative mRNA levels of TNF-α (A) (n = 3). Data are mean±SD. ***P < 0.001. (B) Western blot analysis of protein levels of the indicated proteins in A549 cells with GFP- or viral protein-overexpression.
(TIF)

S8 Fig NS5-M19A is less able to block LCFA β-oxidation and induces less cytokine production. (A) AUC OCR for A549 cells with wild-type NS5 (NS5-WT), M19A-mutated NS5 (NS5-M19A), or vector control were incubated with serum-free medium for 1 h, then treated with PA-BSA or BSA for 18 h (n = 2). (B-D) Cells cultured with serum-free medium for 1 h were incubated with PA-BSA or BSA for 24 h. RT-qPCR analysis of the relative mRNA levels of IL-6 (B) and TNF-α (C) (n = 3). ELISA of the relative protein levels of IL-6 (D) (n = 2). Data are mean±SD. **P < 0.01, and ***P < 0.001.
(TIF)

S9 Fig Interferon (IFN) production and signaling in cells infected with JEV-WT or JEV-NS5-M19A. (A) A549 cells infected with JEV (MOI = 10) for 5 h were replenished with serum-free medium for 1 h, then treated with PA-BSA or BSA for 18 h. RT-qPCR analysis of the relative mRNA levels of interferon β (IFN-β) (n = 3). (B) A549 cells were infected with JEV-WT or JEV-NS5-M19A (MOI = 10) for 24 h in serum-containing medium. RT-qPCR analysis of relative mRNA levels of IFN-β (n = 3). Data are mean±SD. **P < 0.01, ***P < 0.001 and ns, not significant. (C) A549 cells infected with JEV-WT or JEV-NS5-M19A (MOI = 10) for 6 h were stimulated with IFN-αA/D (1000 U/ml) for 30 min or left unstimulated before the cell lysates were harvested for Western blot analysis of p-STAT1, STAT1, NS3 and actin.
(TIF)

S10 Fig Cellular localization of WT and truncated-NS5. (A) Confocal microscopy of pEYFP-Mito-A549 cells transfected with full-length, truncated or mutated NS5 constructs for 24 h before stained with anti-Flag plus Alexa Fluor 568 goat anti-rabbit antibody. (B) The localization of M19 on JEV NS5 protein at two different angles based on the published crystal structure [59].
(TIF)

S11 Fig LCFA β-oxidation and interaction between NS5 and MTP in DENV-2-infected cells. (A) A549 cells infected with JEV or DENV-2 (MOI = 10) for 5 h were replenished with serum-free medium for 1 h, then incubated with PA-BSA or BSA control. AUC OCR measured from 6 to 24 hpi compared to that for mock cells treated with BSA (n = 3). (B) IP with anti-Flag affinity gel and Western blot analysis with the indicated antibodies in HEK293T cells transfected with Flag-tagged JEV NS5 (JNS5) or DENV-2 NS5 (DNS5) for 24 h. (C) Confocal microscopy of pEYFP-Mito-A549 cells transfected with JNS5 or DNS5 plus HADHα-V5-His or HADHβ-HA for 24 h before stained with anti-Flag plus Alexa Fluor 568 goat anti-rabbit or anti-HADHα, anti-V5 or anti-HA plus Alexa Fluor 647 goat anti-mouse antibody.
(TIF)

S12 Fig Effect of etomoxir on JEV replication. (A) A549 cells treated with the indicated doses of etomoxir for 24 h were analyzed by LDH cytotoxicity assay. (B) Fold of β-oxidation was assessed in A549 cells treated with vehicle control or 40 μM etomoxir by XF analyzer (n = 6). Data are mean±SD. ***P < 0.001. (C) A549 cells were pretreated with 40 μM etomoxir for 1 h before virus infection or treated from 4 to 24 hpi (MOI = 5). At 24 hpi, cells were processed for Western blot analysis of protein expression of NS3 and actin.
(TIF)

S13 Fig Activation of AMP-activated kinase (AMPK) by A769662 treatment does not restrict JEV replication. (A) A549 cells treated with A769662 (300 μM) for 12 or 24 h were analyzed for Western blot analysis of protein levels of phospho-AMPK, AMPK and actin. (B and C) A549 cells were treated with or without palmitate (100 μM) for overnight, and then A769662 (300 μM) or solvent control was added 1 h prior JEV infection (MOI = 10 or 0.1). At 24 hpi, cells were processed for immunofluorescent analysis for JEV NS1 (Green) and DAPI (green) (B) and Western blot analysis of protein expression of NS1 and actin (C).
(TIF)

S14 Fig ATP levels in JEV-infected cells cultured with PA-BSA or BSA. A549 cells infected with JEV (MOI = 10) for 5 h were replenished with serum-free medium for 1 h, then cultured with PA-BSA or BSA control. ATP levels of these cells were measured (n = 4). Data are mean±SD. *P < 0.05 and **P < 0.01.
(TIF)
