# Supplementary figures and images for: Japanese Encephalitis Virus Nonstructural Protein NS5 Interacts with Mitochondrial Trifunctional Protein and Impairs Fatty Acid β-Oxidation
Source: PLoS Pathog. 2015 Mar 27;11(3):e1004750. doi: 10.1371/journal.ppat.1004750 (PMC4376648; doi:10.1371/journal.ppat.1004750)

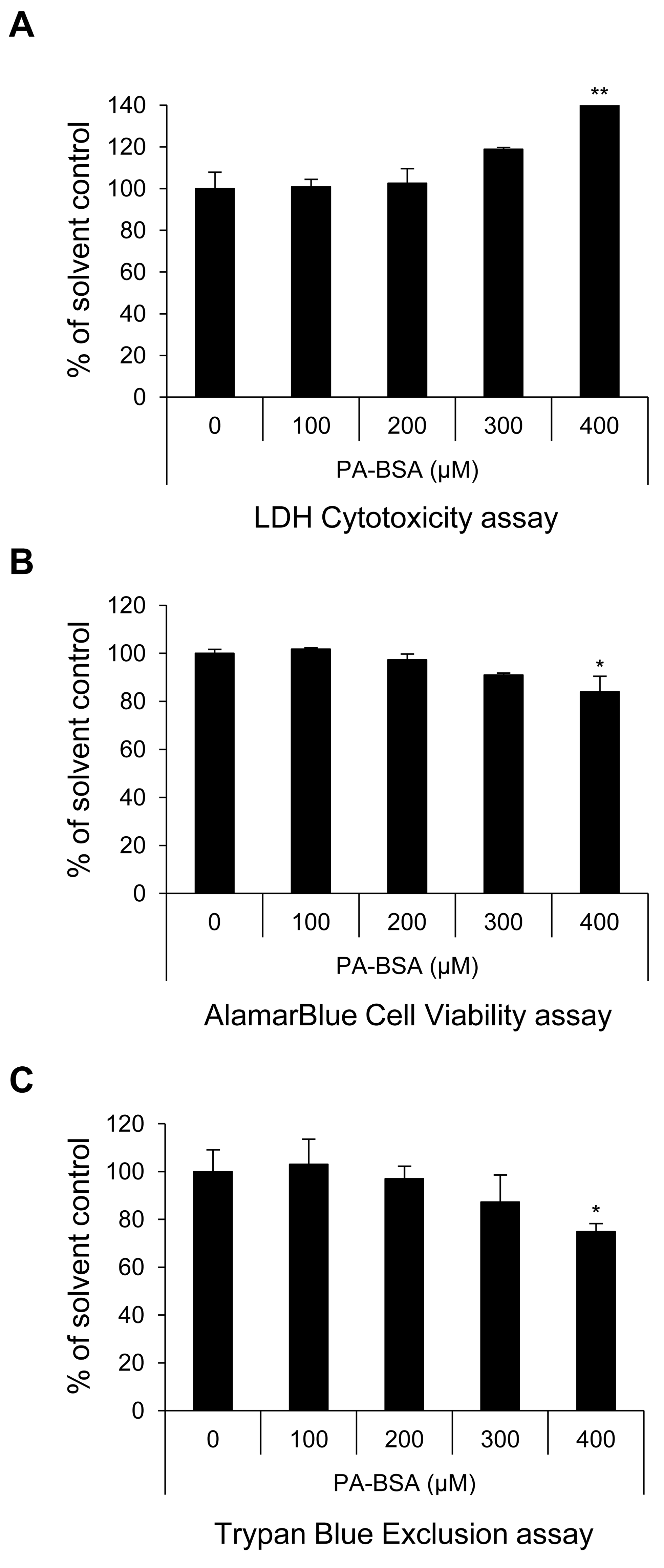

Supplement: S1 Fig — A549 cells were treated with solvent or PA-BSA (up to 400 μM) for 24 h. LDH (A), AlamarBlue (B), and trypan blue exclusion assays (n = 3) (C) were performed to determine cytotoxicity and cell viability. Data are mean±SD. *P < 0.05 and **P < 0.01. (TIF) [file ppat.1004750.s001.tif]

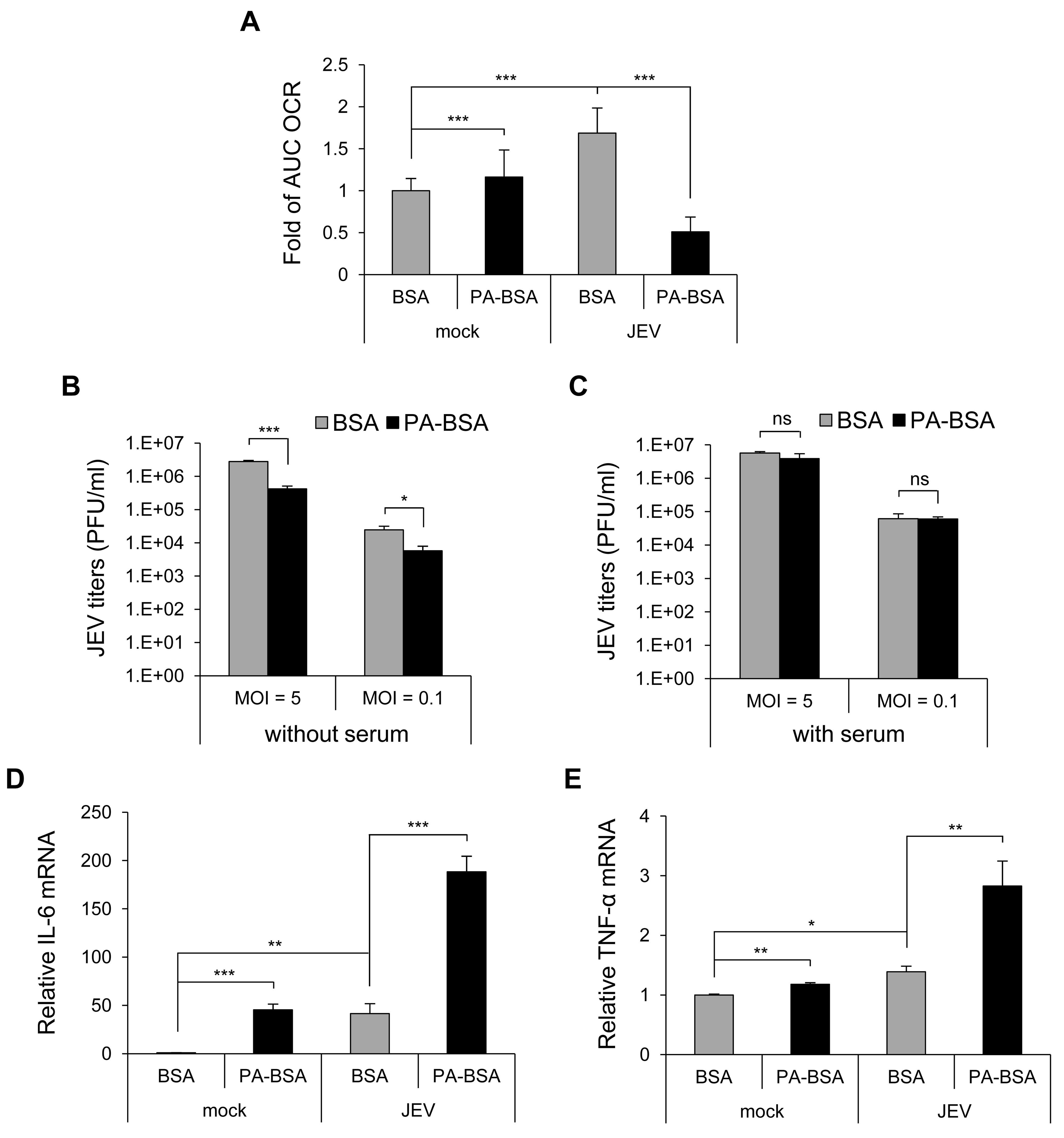

Supplement: S2 Fig — (A) HTB11 cells infected with JEV (MOI = 10) for 5 h were replenished with serum-free medium for 1 h, then incubated with PA-BSA or BSA control. AUC OCR measured from 6 to 24 hpi compared to that for mock cells treated with BSA (n = 2 or 3). (B and C) HTB11 cells infected with JEV (MOI = 5 and 0.1) for 5 h were changed to medium without serum (B) or with serum (10% FBS) (C) for 1 h. Plaque-forming assay of cells treated with PA-BSA or BSA for 18 h before virus titration in culture supernatants (n = 3). (D and E) HTB11 cells infected with JEV (MOI = 10) for 5 h were replenished with serum-free medium for 1 h, then cultured with PA-BSA or BSA control. RT-qPCR analysis of relative mRNA levels of interleukin 6 (IL-6) (D) and tumor necrosis factor α (TNF-α) (E) (n = 3). Data are mean±SD. *P < 0.05, **P < 0.01, ***P < 0.001 and ns, not significant. (TIF) [file ppat.1004750.s002.tif]

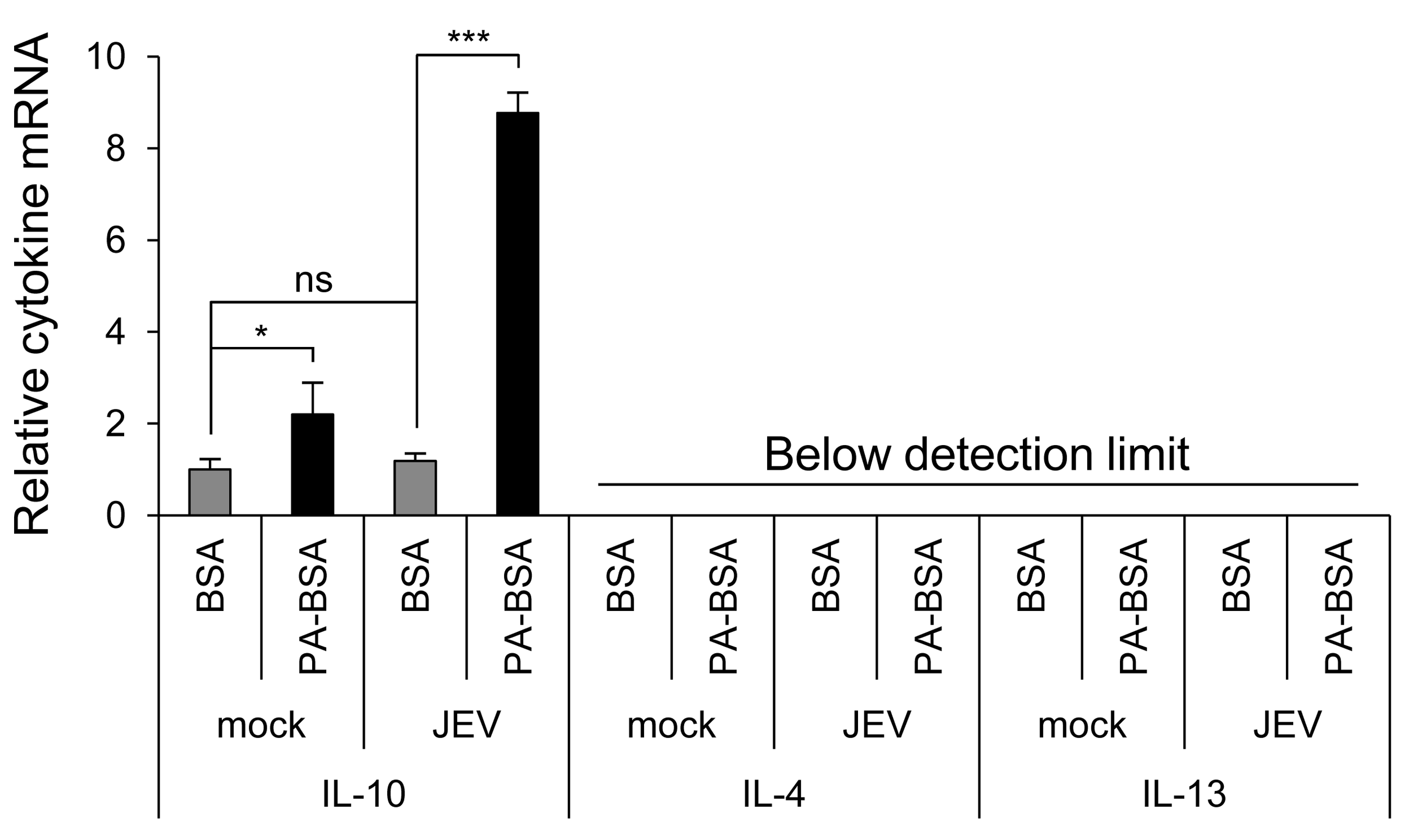

Supplement: S3 Fig — A549 cells infected with JEV (MOI = 10) for 5 h were replenished with serum-free medium for 1 h, then treated with PA-BSA or BSA control for 18 h. RT-qPCR analysis of the relative mRNA levels of IL-10, IL-4 and IL-13 (n = 3). Data are mean±SD. *P < 0.05, ***P < 0.001 and ns, not significant. (TIF) [file ppat.1004750.s003.tif]

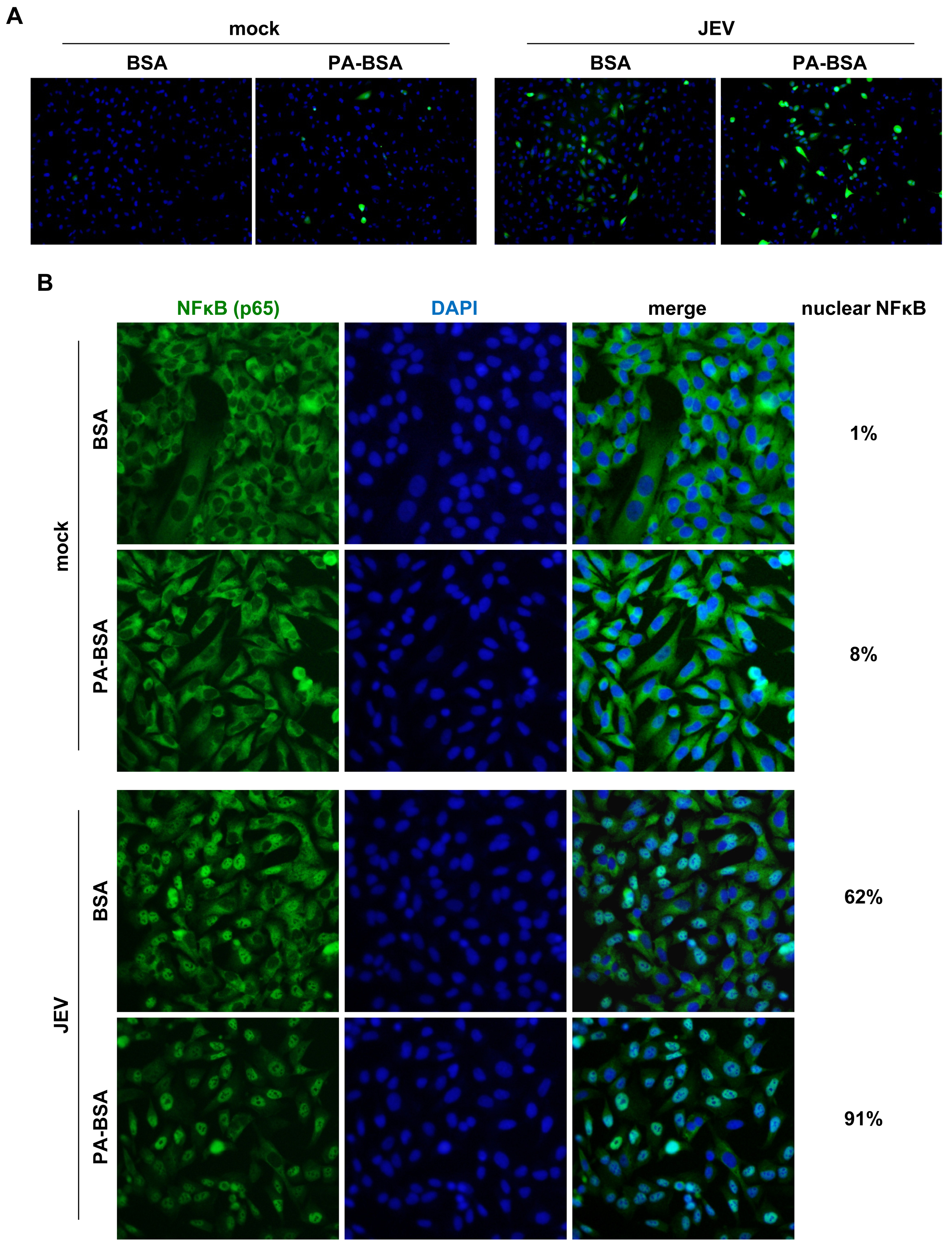

Supplement: S4 Fig — A549 cells infected with JEV (MOI = 10) for 5 h were changed to serum-free medium for 1 h, then treated with PA-BSA or BSA. Fluorescence microscopy of cells stained with DCFH-DA for ROS production represented by green fluorescence (A), or stained with anti-NFκB p65 (green) plus DAPI (blue) (B). (TIF) [file ppat.1004750.s004.tif]

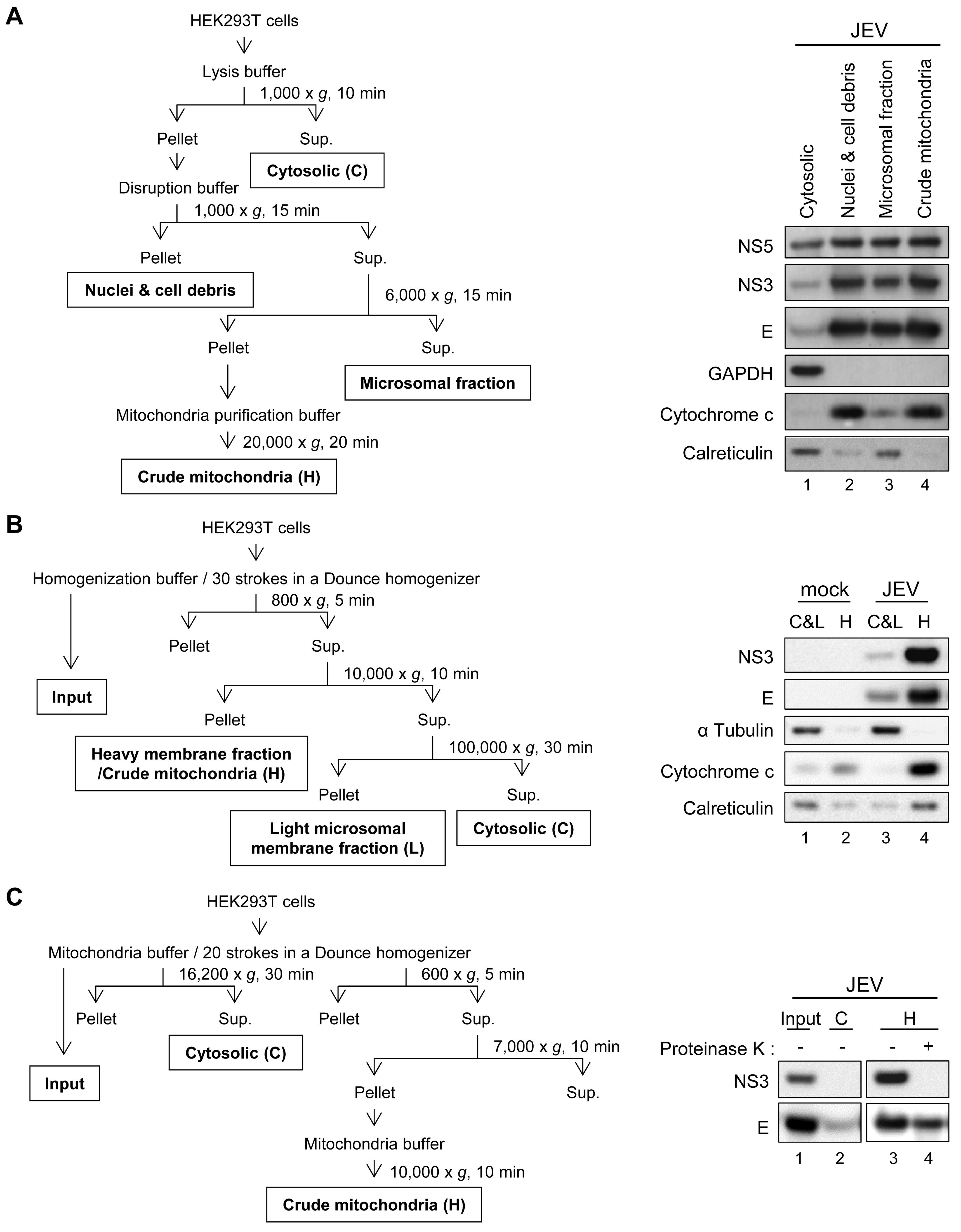

Supplement: S5 Fig — (A) HEK293T cells infected with JEV (MOI = 5) for 24 h were fractionated into cytosolic, nuclei & cell debris, microsomal and crude mitochondria by using Qproteome Mitochondria Isolation Kit. (B and C) Cellular fractions from HEK293T cells infected with JEV (MOI = 3) for 24 h by using the outlined procedure. 10 μg protein per fraction was analyzed by Western blot analysis for the indicated proteins. (C) The mitochondrial fraction isolated from JEV-infected HEK293T cells was treated with or without Proteinase K (100 μg/ml) for 30 min on ice. The reactants were developed by Western blot analysis with antibodies against NS3 and E. C, cytosolic fraction; L, light microsomal membrane fraction; H, heavy membrane fraction/crude mitochondrial fraction. (TIF) [file ppat.1004750.s005.tif]

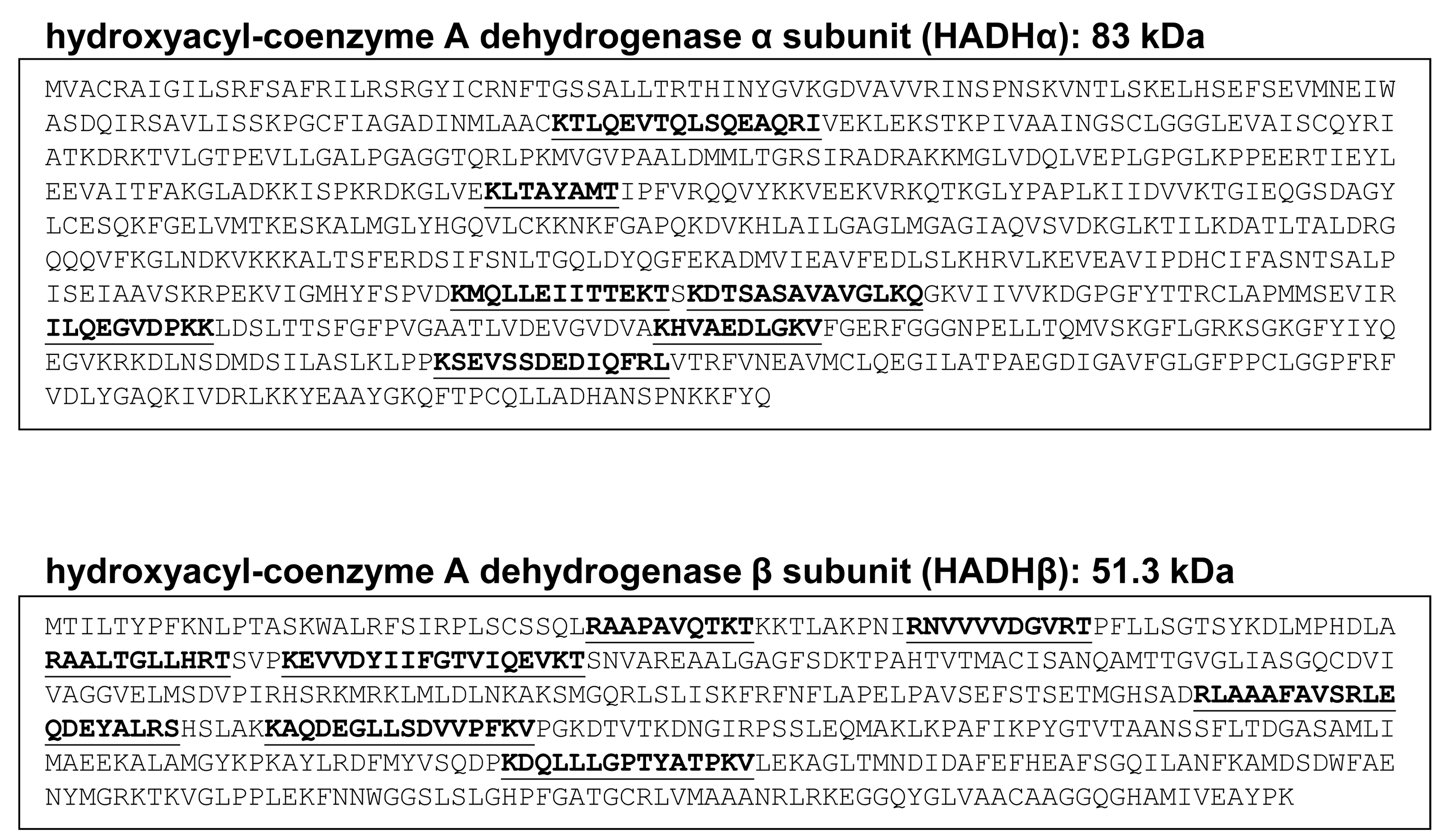

Supplement: S6 Fig — After LC-MS/MS analysis, 83-kDa protein band peptide sequences were matched to HADHα and 51.3-kDa protein band peptide sequences were matched to HADHβ shown in bold and underlined. (TIF) [file ppat.1004750.s006.tif]

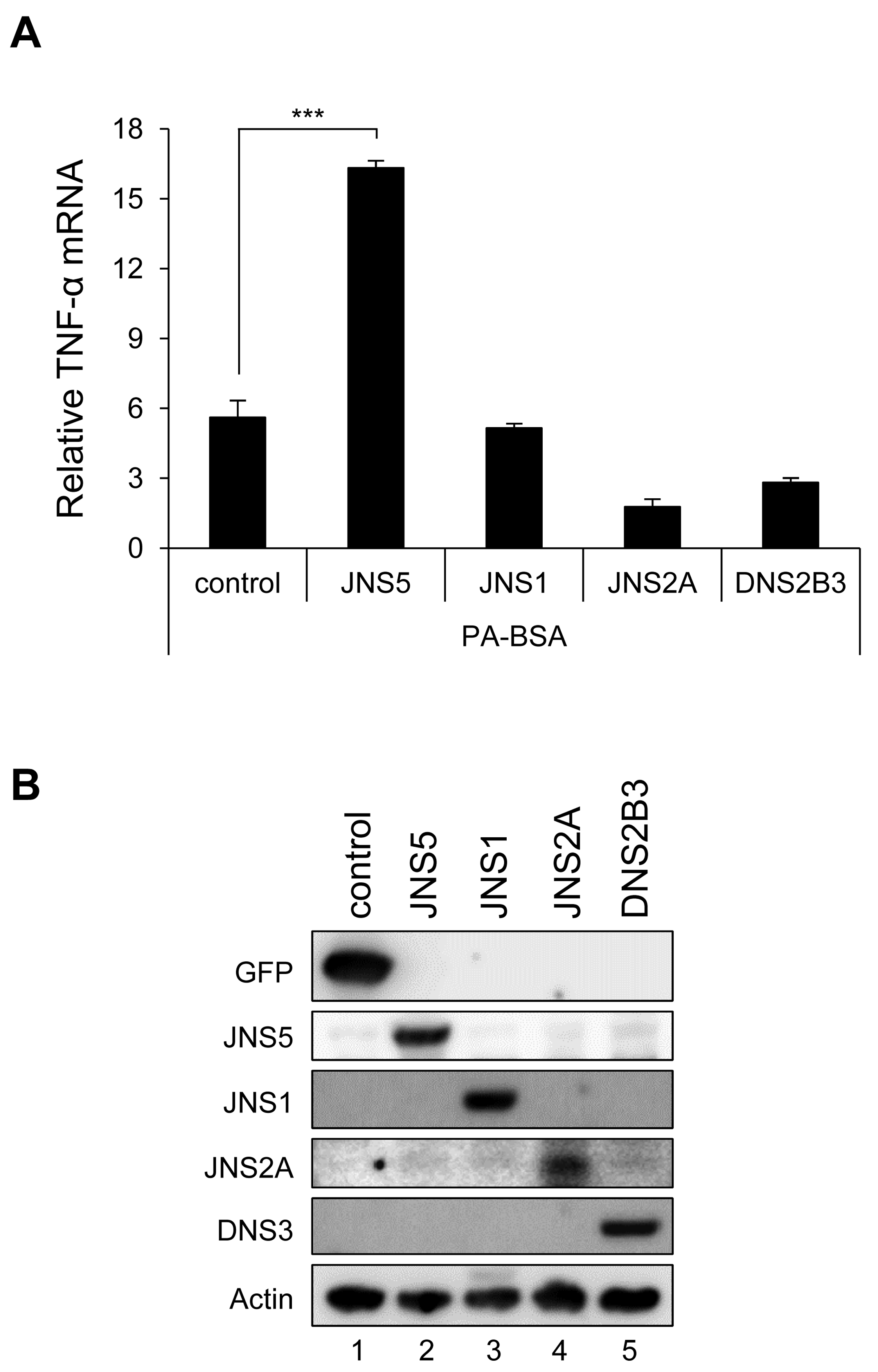

Supplement: S7 Fig — A549 cells with JEV NS5, NS1, NS2A, DENV-2 NS2B3, or GFP control overexpression were cultured with serum-free medium for 1 h, then incubated with medium containing PA-BSA or BSA for 24 h. RT-qPCR analysis of the relative mRNA levels of TNF-α (A) (n = 3). Data are mean±SD. ***P < 0.001. (B) Western blot analysis of protein levels of the indicated proteins in A549 cells with GFP- or viral protein-overexpression. (TIF) [file ppat.1004750.s007.tif]

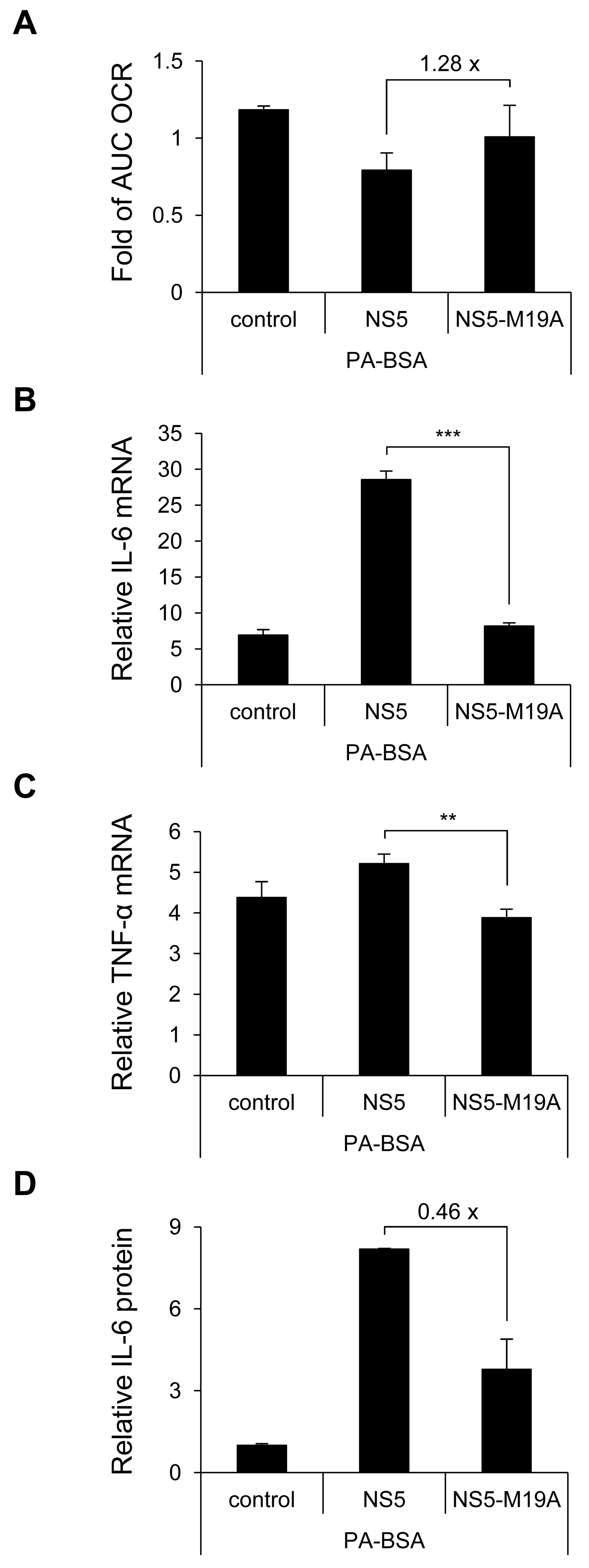

Supplement: S8 Fig — (A) AUC OCR for A549 cells with wild-type NS5 (NS5-WT), M19A-mutated NS5 (NS5-M19A), or vector control were incubated with serum-free medium for 1 h, then treated with PA-BSA or BSA for 18 h (n = 2). (B-D) Cells cultured with serum-free medium for 1 h were incubated with PA-BSA or BSA for 24 h. RT-qPCR analysis of the relative mRNA levels of IL-6 (B) and TNF-α (C) (n = 3). ELISA of the relative protein levels of IL-6 (D) (n = 2). Data are mean±SD. **P < 0.01, and ***P < 0.001. (TIF) [file ppat.1004750.s008.tif]

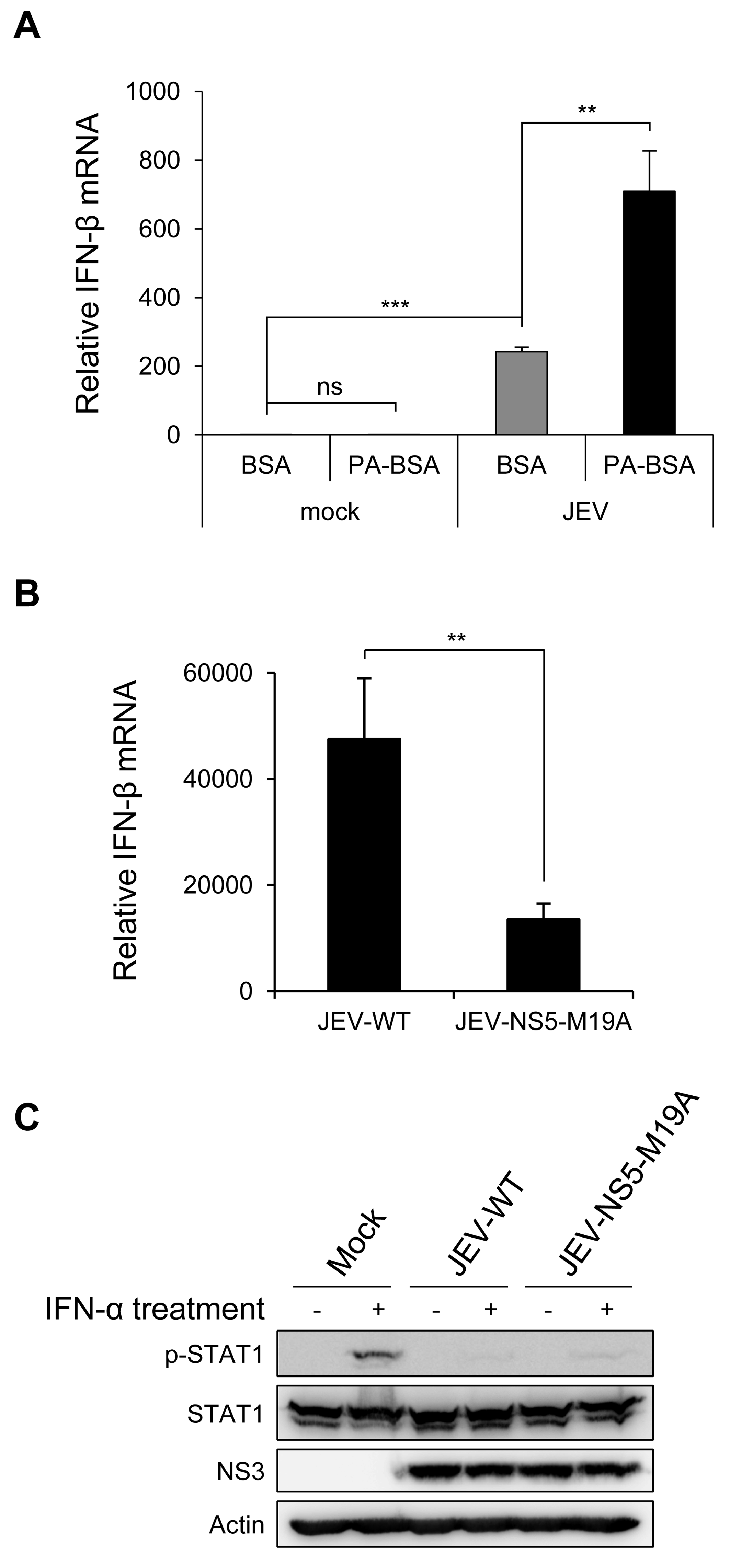

Supplement: S9 Fig — (A) A549 cells infected with JEV (MOI = 10) for 5 h were replenished with serum-free medium for 1 h, then treated with PA-BSA or BSA for 18 h. RT-qPCR analysis of the relative mRNA levels of interferon β (IFN-β) (n = 3). (B) A549 cells were infected with JEV-WT or JEV-NS5-M19A (MOI = 10) for 24 h in serum-containing medium. RT-qPCR analysis of relative mRNA levels of IFN-β (n = 3). Data are mean±SD. **P < 0.01, ***P < 0.001 and ns, not significant. (C) A549 cells infected with JEV-WT or JEV-NS5-M19A (MOI = 10) for 6 h were stimulated with IFN-αA/D (1000 U/ml) for 30 min or left unstimulated before the cell lysates were harvested for Western blot analysis of p-STAT1, STAT1, NS3 and actin. (TIF) [file ppat.1004750.s009.tif]

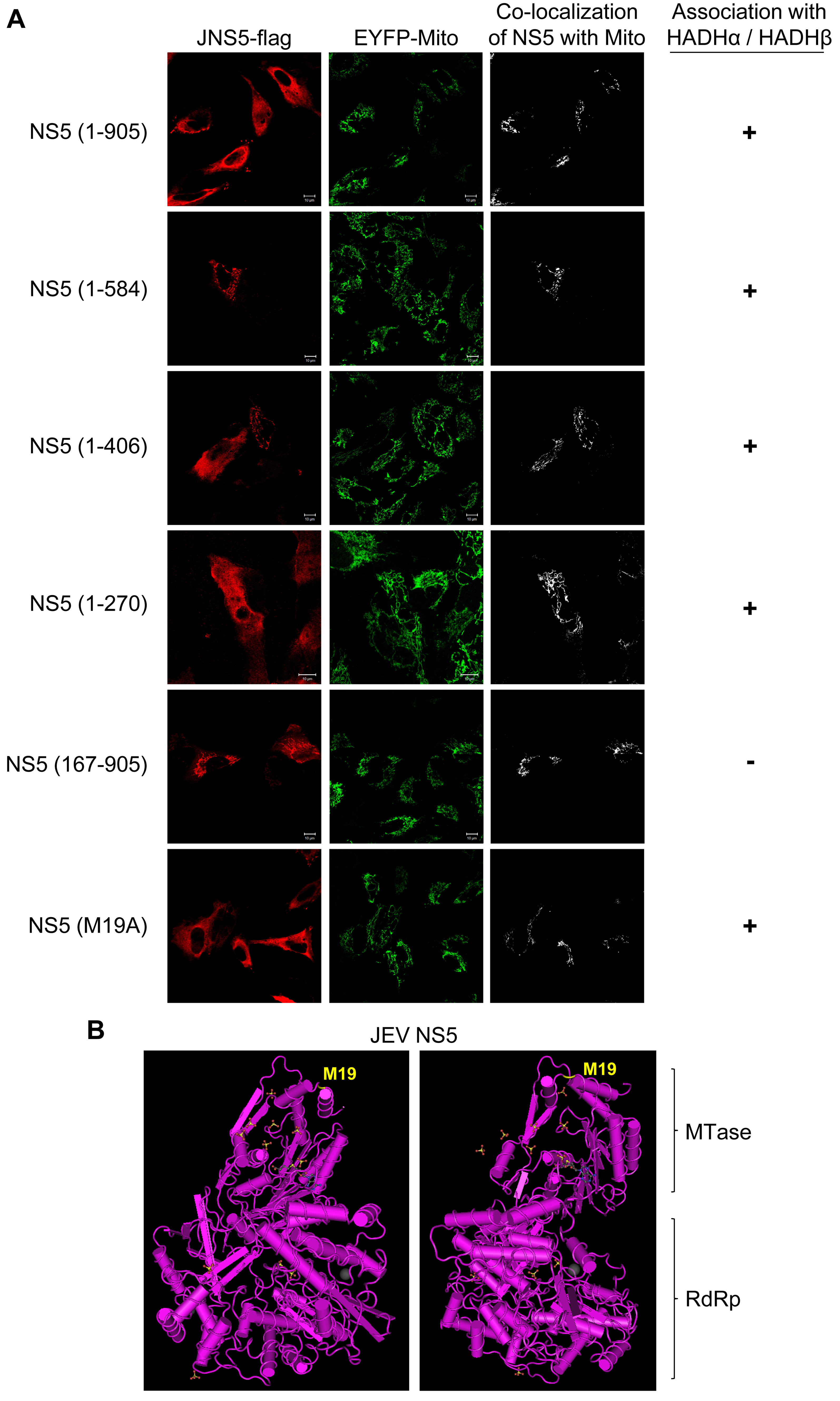

Supplement: S10 Fig — (A) Confocal microscopy of pEYFP-Mito-A549 cells transfected with full-length, truncated or mutated NS5 constructs for 24 h before stained with anti-Flag plus Alexa Fluor 568 goat anti-rabbit antibody. (B) The localization of M19 on JEV NS5 protein at two different angles based on the published crystal structure [59]. (TIF) [file ppat.1004750.s010.tif]

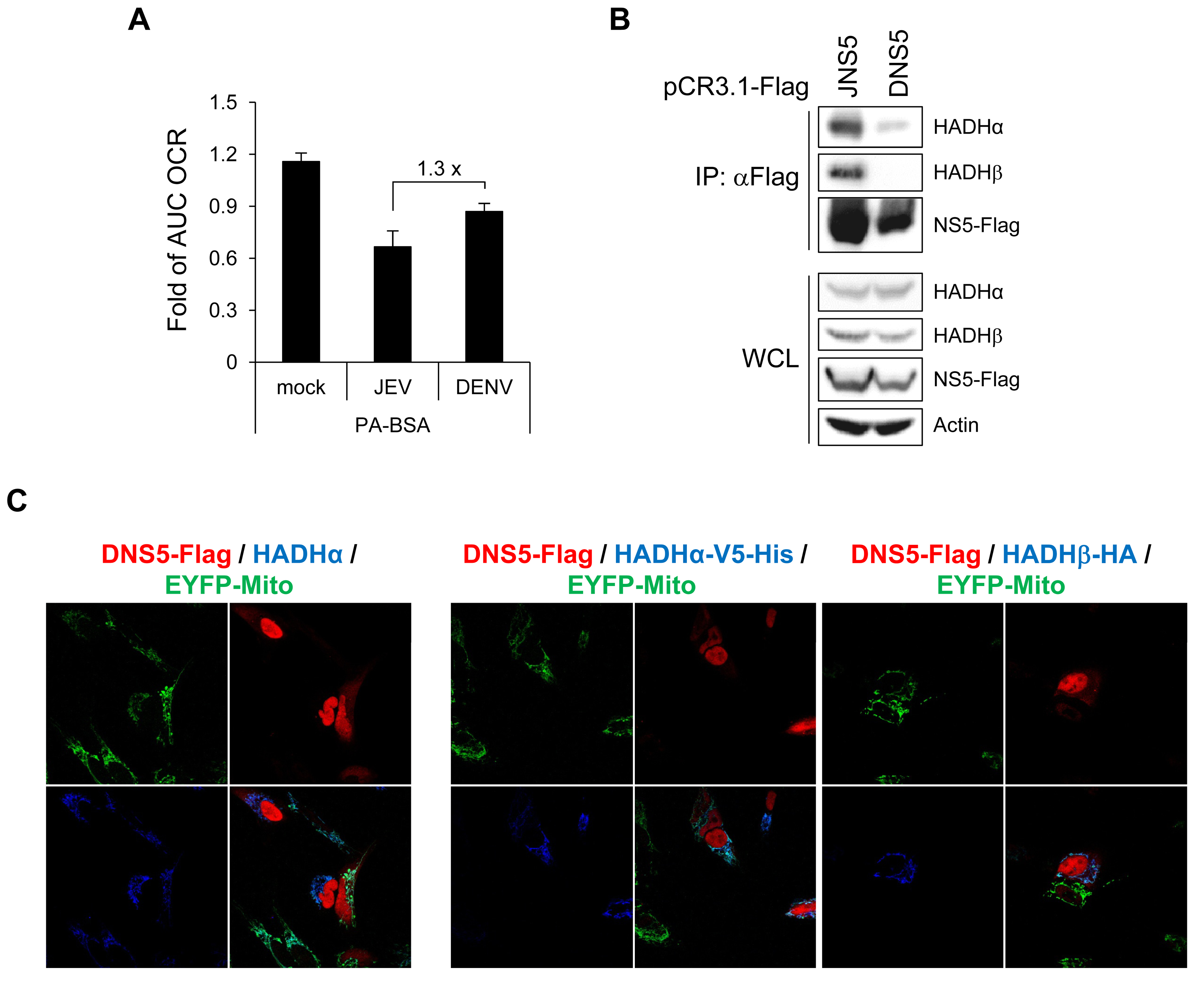

Supplement: S11 Fig — (A) A549 cells infected with JEV or DENV-2 (MOI = 10) for 5 h were replenished with serum-free medium for 1 h, then incubated with PA-BSA or BSA control. AUC OCR measured from 6 to 24 hpi compared to that for mock cells treated with BSA (n = 3). (B) IP with anti-Flag affinity gel and Western blot analysis with the indicated antibodies in HEK293T cells transfected with Flag-tagged JEV NS5 (JNS5) or DENV-2 NS5 (DNS5) for 24 h. (C) Confocal microscopy of pEYFP-Mito-A549 cells transfected with JNS5 or DNS5 plus HADHα-V5-His or HADHβ-HA for 24 h before stained with anti-Flag plus Alexa Fluor 568 goat anti-rabbit or anti-HADHα, anti-V5 or anti-HA plus Alexa Fluor 647 goat anti-mouse antibody. (TIF) [file ppat.1004750.s011.tif]

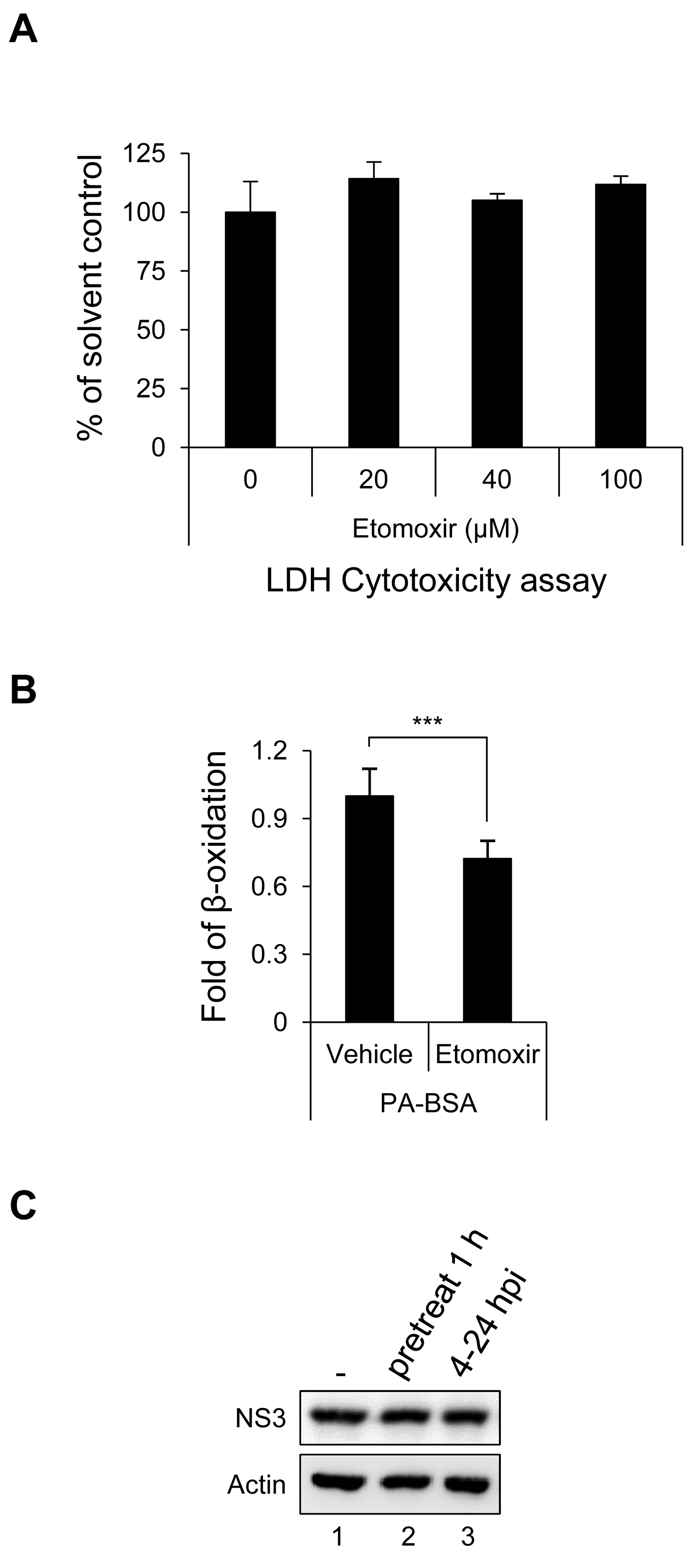

Supplement: S12 Fig — (A) A549 cells treated with the indicated doses of etomoxir for 24 h were analyzed by LDH cytotoxicity assay. (B) Fold of β-oxidation was assessed in A549 cells treated with vehicle control or 40 μM etomoxir by XF analyzer (n = 6). Data are mean±SD. ***P < 0.001. (C) A549 cells were pretreated with 40 μM etomoxir for 1 h before virus infection or treated from 4 to 24 hpi (MOI = 5). At 24 hpi, cells were processed for Western blot analysis of protein expression of NS3 and actin. (TIF) [file ppat.1004750.s012.tif]

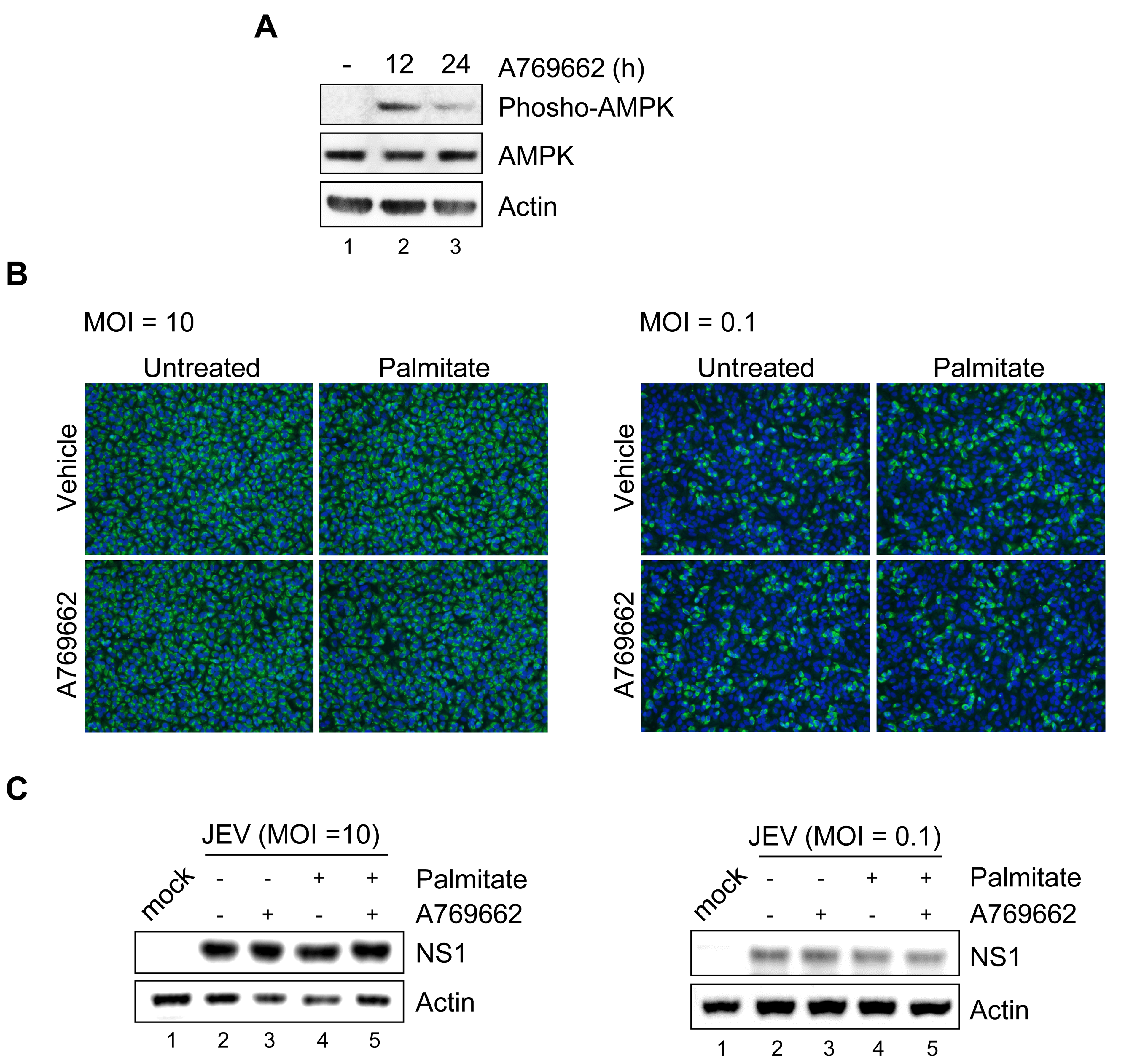

Supplement: S13 Fig — (A) A549 cells treated with A769662 (300 μM) for 12 or 24 h were analyzed for Western blot analysis of protein levels of phospho-AMPK, AMPK and actin. (B and C) A549 cells were treated with or without palmitate (100 μM) for overnight, and then A769662 (300 μM) or solvent control was added 1 h prior JEV infection (MOI = 10 or 0.1). At 24 hpi, cells were processed for immunofluorescent analysis for JEV NS1 (Green) and DAPI (green) (B) and Western blot analysis of protein expression of NS1 and actin (C). (TIF) [file ppat.1004750.s013.tif]

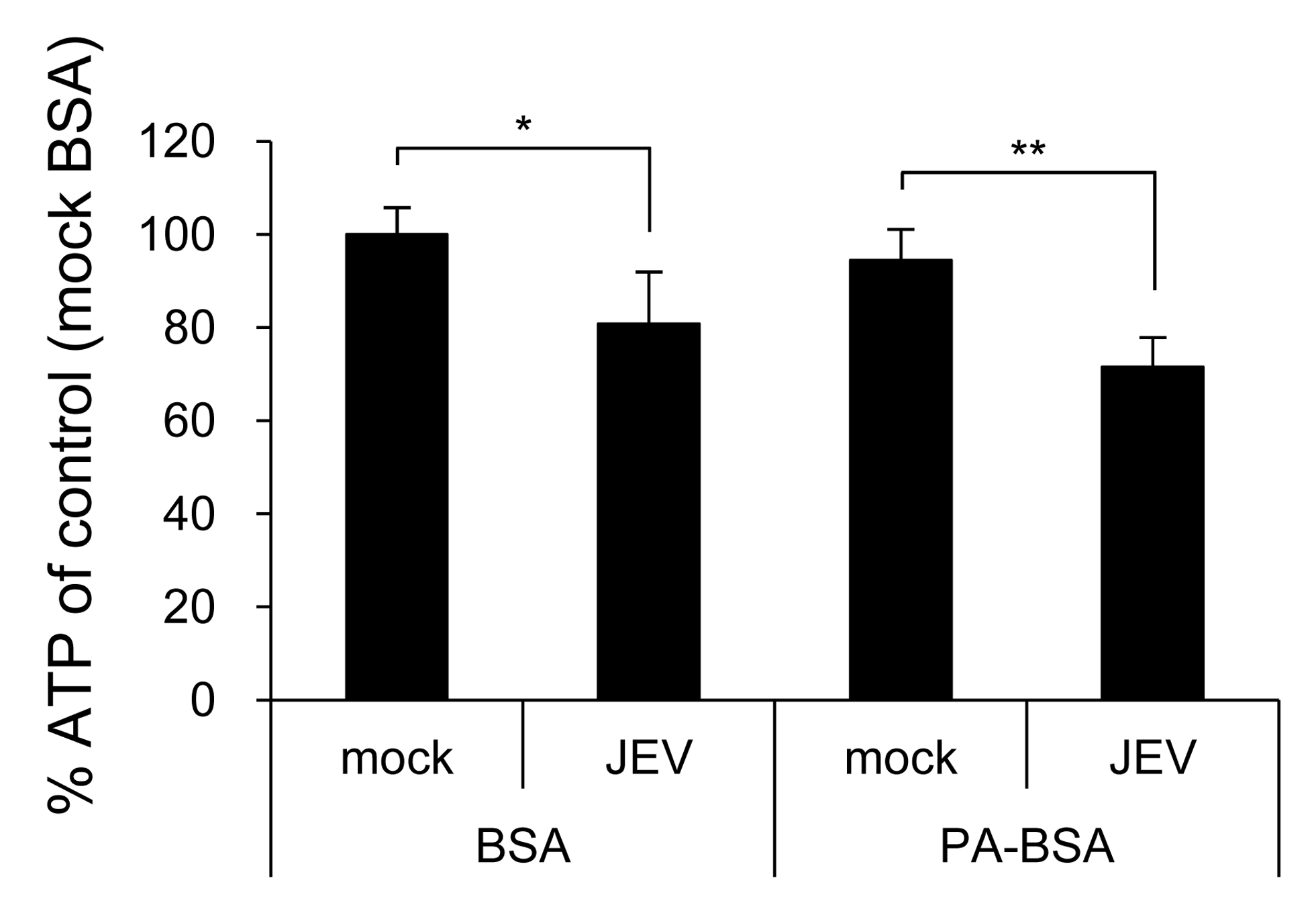

Supplement: S14 Fig — A549 cells infected with JEV (MOI = 10) for 5 h were replenished with serum-free medium for 1 h, then cultured with PA-BSA or BSA control. ATP levels of these cells were measured (n = 4). Data are mean±SD. *P < 0.05 and **P < 0.01. (TIF) [file ppat.1004750.s014.tif]
